# The deubiquitinase CYLD is a specific checkpoint of the STING antiviral signaling pathway

**DOI:** 10.1371/journal.ppat.1007435

**Published:** 2018-11-02

**Authors:** Lele Zhang, Ning Wei, Ye Cui, Ze Hong, Xing Liu, Qiang Wang, Senlin Li, Heng Liu, Huansha Yu, Yanni Cai, Quanyi Wang, Juanjuan Zhu, Wei Meng, Zhengjun Chen, Chen Wang

**Affiliations:** 1 State Key Laboratory of Cell Biology, CAS Center for Excellence in Molecular Cell Science, Shanghai Institute of Biochemistry and Cell Biology, Chinese Academy of Sciences, University of Chinese Academy of Sciences, Shanghai, China; 2 State Key Laboratory of Natural Medicines, School of Life Science and Technology, China Pharmaceutical University, 639 Longmian Avenue, Jiangning District, Nanjing, China; 3 Key Laboratory of Molecular Virology and Immunology, Institut Pasteur of Shanghai, Chinese Academy of Sciences, Shanghai, China; 4 School of Life Science and Technology, Shanghai Tech University, Shanghai, China; Emory Vaccine Center, UNITED STATES

## Abstract

Stimulator of interferon genes (STING) is critical for cytosolic DNA-triggered innate immunity. STING is modified by several types of polyubiquitin chains. Here, we report that the deubiquitinase CYLD sustains STING signaling by stabilizing the STING protein. CYLD deficiency promoted the K48-linked polyubiquitination and degradation of STING, attenuating the induction of IRF3-responsive genes after HSV-1 infection or the transfection of DNA ligands. Additionally, CYLD knockout mice were more susceptible to HSV-1 infection than their wild-type (WT) littermates. Mechanistically, STING translocated from the ER to the Golgi upon HSV-1 stimulation; CYLD partially accumulated with STING and interacted selectively with K48-linked polyubiquitin chains on STING, specifically removing the K48-linked polyubiquitin chains from STING and ultimately boosting the innate antiviral response. Our study reveals that CYLD is a novel checkpoint in the cGAS-STING signaling pathway and sheds new light on the dynamic regulation of STING activity by ubiquitination.

## Author summary

STING is critical for mediating the production of type I interferons and other proinflammatory cytokines. The appropriate activation of STING signaling is precisely modulated to maintain immune homeostasis. It is well established that covalent modification of STING by different types of polyubiquitin chains serves to fine-tune STING activity in response to extracellular and intracellular stresses. However, it remains poorly understood how these polyubiquitin chains on STING are dynamically removed in response to different stimuli. In this study, we characterized the deubiquitinase CYLD, which partially accumulates with STING upon HSV-1 infection and interacts selectively with the K48-linked polyubiquitin chains on STING. CYLD specifically removes K48-linked polyubiquitin chains from STING and thus promotes antiviral responses. Our study reveals a novel function of CYLD in the STING signaling pathway and indicates that CYLD is an important target for modulating the host response to infections caused by DNA pathogens.

## Introduction

The innate immune system represents the first line of host defense against invading pathogens and employs germline-encoded pattern-recognition receptors (PRRs) to detect conserved microbial molecules known as pathogen-associated molecular patterns (PAMPs). Upon sensing their corresponding PAMPs, PRRs activate signaling cascades that trigger the expression of downstream genes, which collaboratively restrain microbes and activate adaptive immune responses [1].

RIG-I and MDA5 detect cytosolic RNAs and recruit mitochondrial MAVS, which activate TBK1 and IKK kinases to phosphorylate the transcription factors IRF3 and NF-κB, ultimately inducing the expression of type I interferons (IFNs) and proinflammatory cytokines [2,3]. Cytosolic aberrant DNAs are potentially sensed by cyclic GMP-AMP synthase (cGAS), DNA-dependent activator of IFN-regulatory factors (DAI), DEAD-box helicase 41 (DDX41) or interferon gamma inducible protein 16 (IFI16) [4–11]. Interestingly, the signaling pathways triggered by these sensors all converge at stimulator of interferon genes (STING, also known as MITA, ERIS or MPYS), an endoplasmic reticulum (ER) protein [12–15] that recruits and activates TBK1 and IKK to ultimately induce the expression of antiviral and proinflammatory genes.

The action of STING is extensively regulated by various types of ubiquitin modifications [16]. For example, the E3 ubiquitin ligases RNF5 and TRIM29 catalyze K48-linked polyubiquitination of STING, facilitating its proteasome-mediated degradation [17,18]. TRIM56 and TRIM32 target STING for K63-linked polyubiquitination [19,20] and enhance the downstream signaling. AMFR/INSIG1 catalyze the K27-linked polyubiquitination of STING, which is crucial for recruiting and activating TBK1 [21]. RNF26 promotes K11-linked polyubiquitination of STING at lysine 150, a residue that is also targeted by RNF5 for K48-linked polyubiquitination [22]. Notably, it remains largely unknown how polyubiquitin chains on STING are dynamically removed in response to different stimuli.

CYLD was identified as a tumor suppressor that is mutated in familial cylindromatosis, and it plays important roles in development and tumorigenesis [23–26]. Biochemically, CYLD is a deubiquitinase that can remove K63- or K48-linked polyubiquitin chains in a reaction catalyzed by its C-terminal USP domain [27–31]. A recent study reported that SDC4 could recruit CYLD to remove polyubiquitin chains from RIG-I, thus inhibiting RIG-I signaling [32]. The potential role of CYLD in the STING signaling pathway remains to be explored.

In this study, we demonstrated that silencing CYLD markedly attenuated the STING-mediated induction of antimicrobial genes. Consistent with this finding, CYLD knockout mice were more susceptible to HSV-1 infection than their WT littermates. Mechanistically, CYLD partially accumulated with STING upon HSV-1 stimulation and interacted selectively with K48-linked polyubiquitin chains on STING. CYLD specifically removed K48-linked polyubiquitin chains from STING and ultimately boosted host innate antiviral responses. This study uncovers an essential function of CYLD in the STING signaling pathway and provides a new perspective for restricting infections caused by DNA pathogens.

## Results

### CYLD is a new regulator of DNA-triggered antiviral signaling pathway

**Fig 1:**
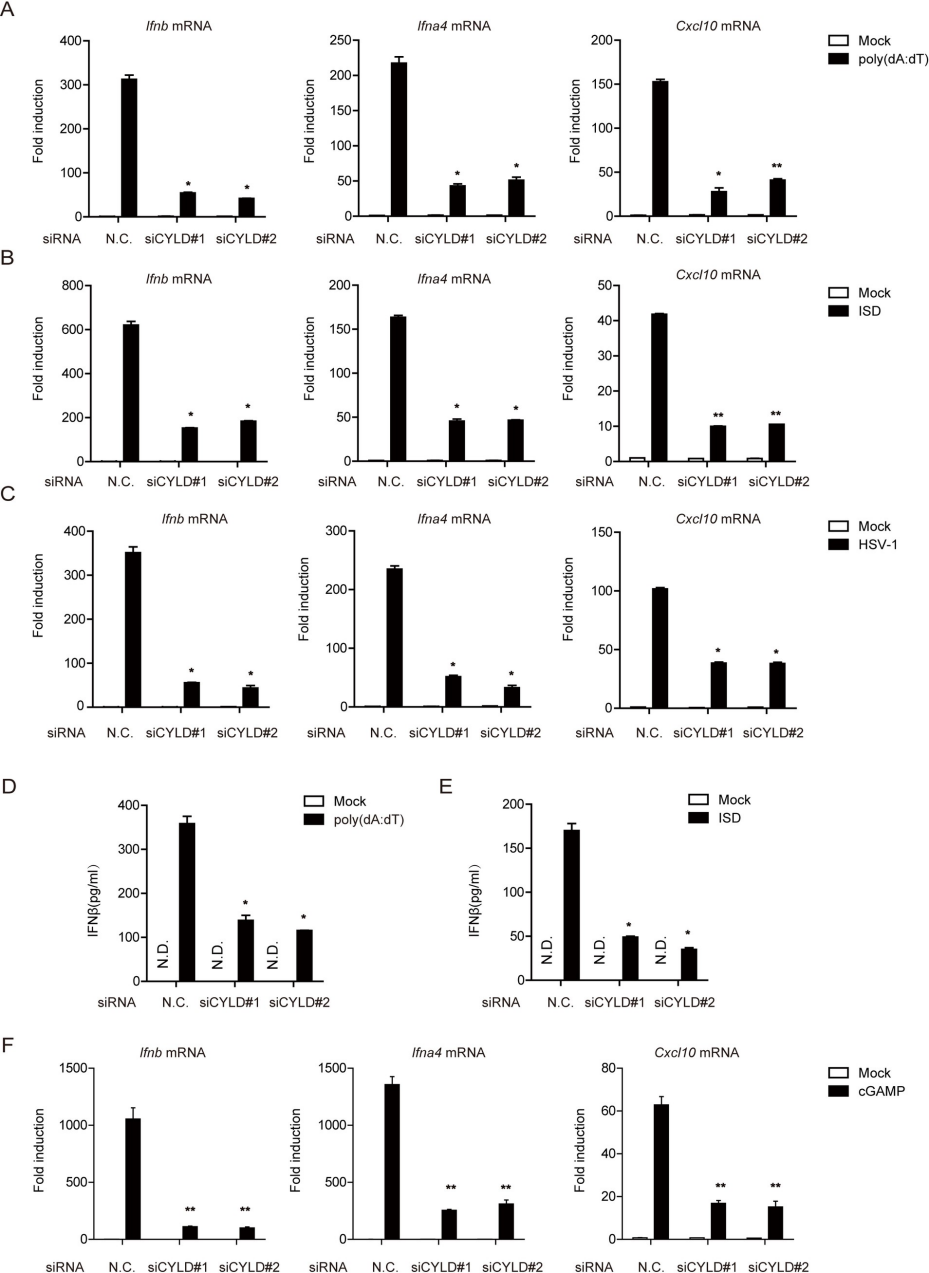
Identification of CYLD as a new regulator of the DNA-triggered signaling pathway. (**A**, **B** and **C**) MEFs transfected with negative control (N.C.) or CYLD siRNAs were stimulated with poly(dA:dT) (3 μg per well), ISD (5 μg per well) or HSV-1 (MOI = 1) for 6 h. Then, the induction of *Ifnb*, *Ifna4*, and *Cxcl10* mRNAs was measured by quantitative PCR. (**D** and **E**) MEFs transfected with the indicated siRNA were stimulated with poly(dA:dT) (3 μg per well) or ISD (5 μg per well) for 6 h. IFN-β production was assayed by ELISA. (**F**) MEFs transfected with negative control (N.C.) or CYLD siRNAs were incubated with or without cGAMP (1 μg per well) for 30 minutes at 37°C, and the medium was then replaced. After cGAMP delivery for the indicated time periods, the induction of *Ifnb* and *Ifna4* mRNAs was measured by quantitative PCR. Graphs show the mean ± s.d., and the data (**A**-**F**) shown are representative of three independent experiments. *P <0.05; **P <0.01 (two-tailed t-test).

To explore the potential function of CYLD in STING signaling, we screened two different siRNAs against murine CYLD (siCYLD#1 and siCYLD#2 for mouse). Knockdown of *Cyld* significantly inhibited the expression of IRF3-responsive genes (*Ifnb*, *Ifna4*, and *Cxcl10*) induced by either poly(dA:dT) or interferon stimulatory DNA (ISD) (a cytosolic DNA mimic) in mouse embryonic fibroblasts (MEFs) (Fig 1A and 1B). Moreover, silencing of *Cyld* markedly attenuated antiviral gene expression upon HSV-1 infection, as shown in Fig 1C. Furthermore, we measured the secretion of IFN-β. Consistent with the above results, *Cyld* knockdown in MEFs resulted in decreased production of IFN-β upon poly(dA:dT) and ISD stimulation (Fig 1D and 1E). In addition, the expression of IRF3-responsive genes triggered by cyclic GMP-AMP (cGAMP), a ligand that specifically activates STING, was markedly decreased in *Cyld*-silenced MEFs (Fig 1F). We also observed that silencing of *Cyld* increased the expression of IRF3-responsive genes induced by poly(I:C) (S1A Fig), which is consistent with the results of previous studies [32,33]. The efficiency of siRNA-mediated knockdown of *Cyld* mRNA is shown in S1B Fig. Collectively, these data suggest that CYLD is a new positive regulator of the DNA-triggered signaling pathway.

### The deubiquitylation activity of CYLD is essential for potentiating DNA-triggered signaling

**Fig 2:**
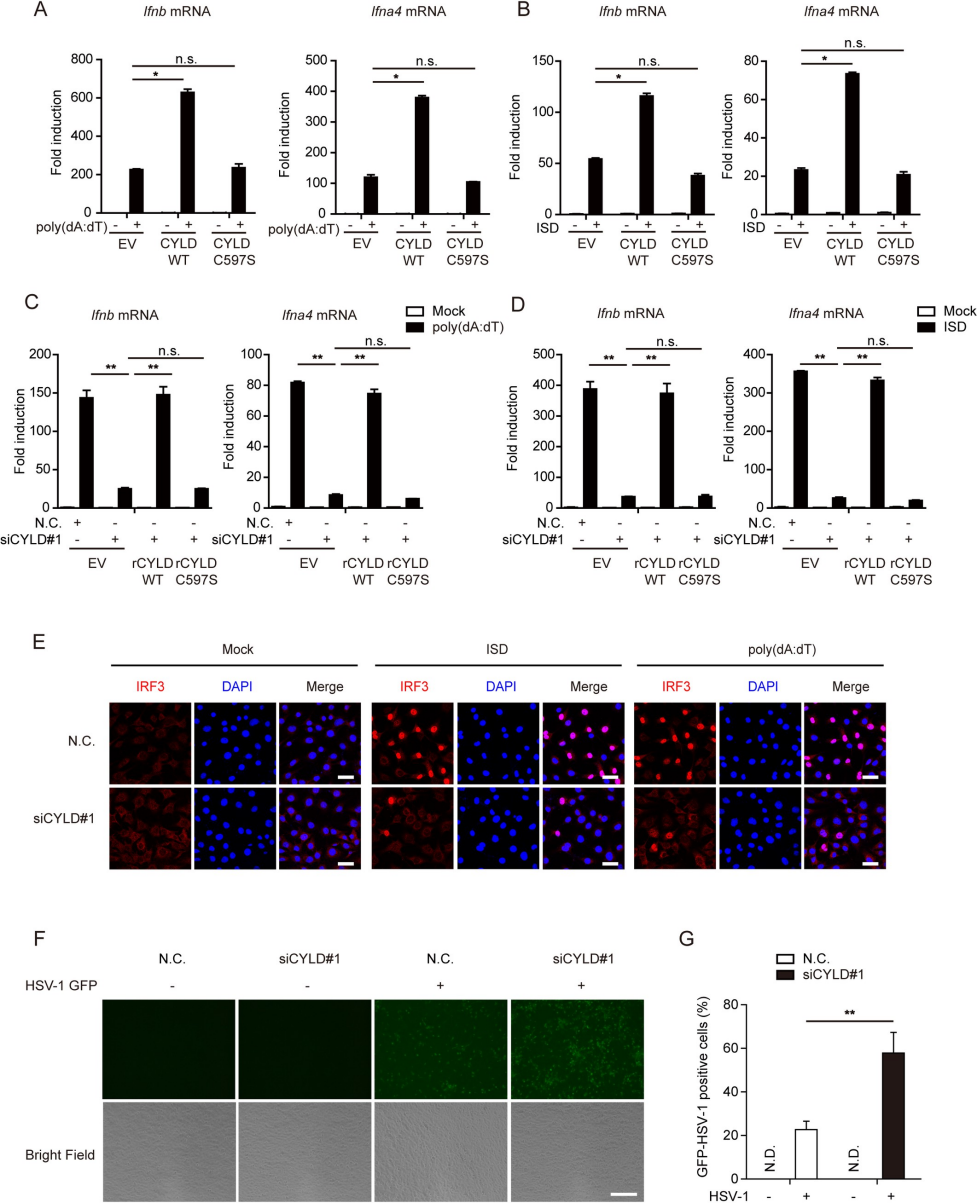
CYLD is essential for potentiating DNA-triggered signaling. (**A** and **B**) MEFs were transfected with 1 μg of empty vector (EV) or plasmids for the expression of wild-type HA-CYLD or HA-CYLD C597S. Then, the induction of *Ifnb* and *Ifna4* mRNAs was measured by quantitative PCR after poly(dA:dT) (3 μg per well) or ISD (5 μg per well) stimulation for 6 h. (**C** and **D**) MEFs were transfected with 2 μl of negative control (N.C.) or CYLD siRNA#1 for 24 h and then transfected with the indicated siRNA-resistant constructs for another 24 h, followed by stimulation with poly(dA:dT) (3 μg per well) or ISD (5 μg per well) for 6 h. Then, the induction of *Ifnb* and *Ifna4* mRNAs was measured by quantitative PCR. (**E**) MEFs (12-well plate) transfected with negative control (N.C.) or CYLD siRNA#1 were stimulated with poly(dA:dT) (3 μg per well) or ISD (5 μg per well) for 4 h, then stained with an antibody specific for IRF3 and imaged by confocal microscopy. Scale bars represent 50 μm. (**F** and **G**) MEFs (12-well plate) were transfected with the negative control (N.C.) or CYLD siRNA#1 and infected with HSV-1-GFP (MOI = 1) 24 h later. GFP expression was visualized 16 h later by fluorescence microscopy (scale bars represent 100 μm) (**F**) and quantified (**G**) from five independent experiments. Graphs show the mean ± s.d., and the data (**A-G**) shown are representative of three independent experiments. n.s., not significant; *P <0.05; **P <0.01 (two-tailed t-test).

Given that CYLD is a deubiquitinase, we investigated whether its effect was dependent on its deubiquitylation activity. We constructed a mouse CYLD C597S mutant (cysteine mutated to serine at position 597), which lacked deubiquitination activity (S2E Fig) [34]. As expected, ectopic expression of CYLD WT enhanced poly(dA:dT)- or ISD- induced IFN gene expression. In contrast, ectopic expression of the CYLD C597S mutant failed to exert the same effect (Figs 2A, 2B, S2A and S2B).

To rule out potential off-target effects of CYLD siRNAs, we generated two RNA interference (RNAi)-resistant CYLD constructs, namely, rCYLD WT and rCYLD C597S, in which silent mutations were introduced into the sequence targeted by the siRNA without changing the amino acid sequence of the corresponding protein. As shown in Figs 2C, 2D, S2C and S2D, the diminished IFN production in *Cyld****-***silenced cells was restored by introducing rCYLD WT but not rCYLD C597S after stimulation with poly(dA:dT) or ISD. Substantiating this finding, the nuclear translocation of IRF3 was markedly reduced when *Cyld* was knocked down in MEFs (Figs 2E and S2F). Furthermore, knocking down *Cyld* facilitated HSV-1-GFP virus infection, as evidenced by stronger GFP-positive signals (Fig 2F and 2G). Next, *Cyld****-***silenced MEFs or control MEFs were challenged with HSV-1, and the titers of HSV-1 were analyzed by a standard plaque assay. Consistent with the above findings, *Cyld* knockdown resulted in a marked increase in viral titers compared with those of the controls, as shown in S2G Fig. Taken together, these results suggest that the deubiquitylation activity of CYLD is indispensable for regulating the DNA-triggered signaling pathway.

### CYLD deficiency impairs the DNA-triggered signaling pathway

**Fig 3:**
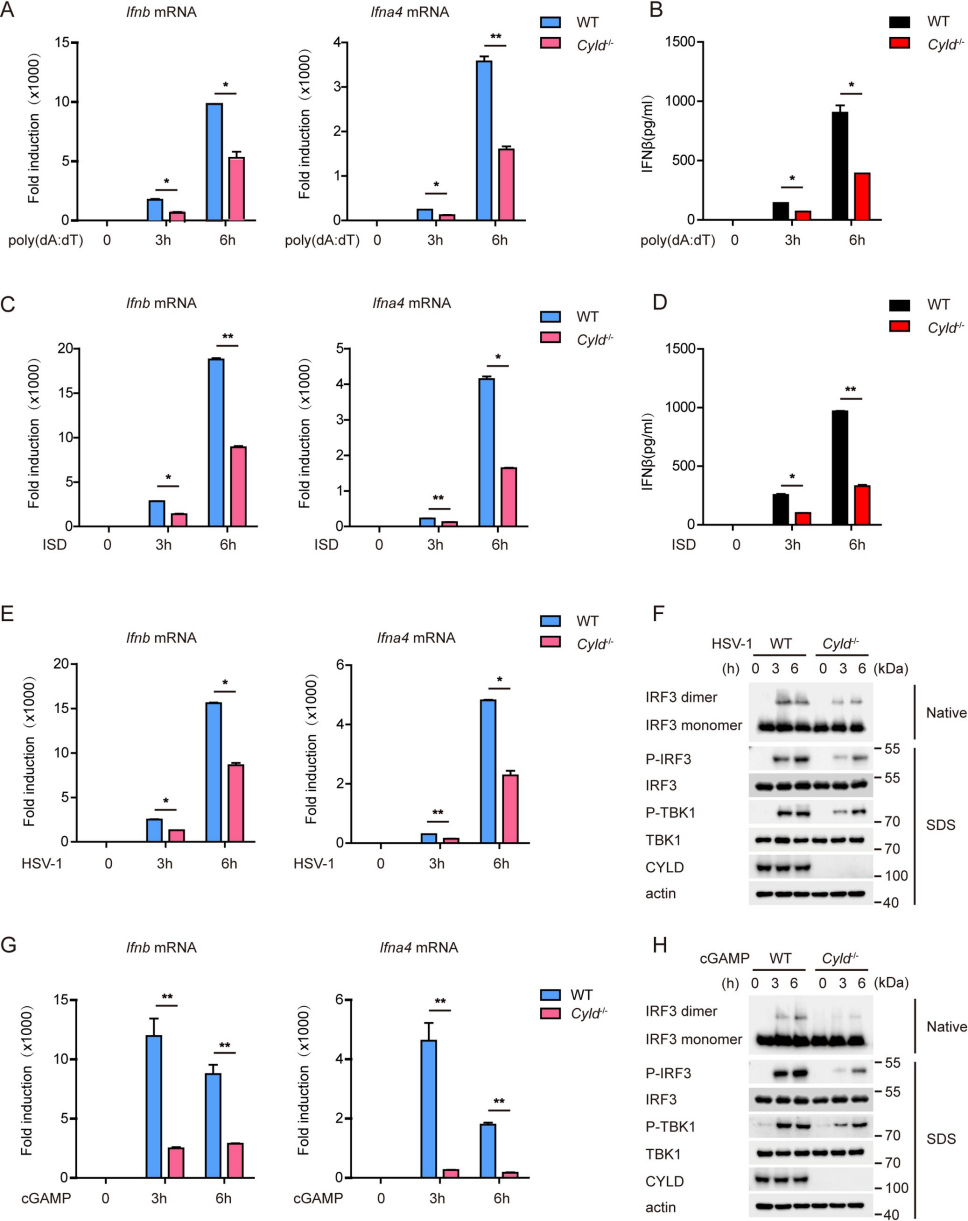
CYLD deficiency impairs the DNA-triggered type I IFN signaling pathway. (**A**) WT and *Cyld*-deficient BMDMs were stimulated with mock or poly(dA:dT) (3 μg per well) for the indicated time periods. The induction of *Ifnb* and *Ifna4* mRNAs was measured by quantitative PCR. (**B**) WT or *Cyld*-deficient BMDMs were treated as described in (**A**), and IFN-β production was determined by ELISA. (**C**) WT and *Cyld*-deficient BMDMs were stimulated with mock or ISD (5 μg per well) for the indicated time periods. The induction of *Ifnb* and *Ifna4* mRNAs was measured by quantitative PCR. (**D**) WT or *Cyld*-deficient BMDMs were treated as described in (**C**), and IFN-β production was determined by ELISA. (**E**) WT and *Cyld*-deficient BMDMs were mock infected or infected with HSV-1 (MOI = 5) for the indicated time periods. The induction of *Ifnb* and *Ifna4* mRNAs was measured by quantitative PCR. (**F**) WT and *Cyld*-deficient BMDMs were mock infected or infected with HSV-1 (MOI = 5) for the indicated time periods, and cell extracts were analyzed for IRF3 dimerization and for IRF3 phosphorylation and TBK1 phosphorylation by native PAGE and SDS-PAGE, respectively. (**G**) WT and *Cyld*-deficient BMDMs were incubated with or without cGAMP (1 μg per well) for 30 minutes at 37°C, and the medium was then replaced. After cGAMP delivery for the indicated time periods, the induction of *Ifnb* and *Ifna4* mRNAs was measured by quantitative PCR. (**H**) WT and *Cyld*-deficient BMDMs were incubated with or without cGAMP (1 μg per well) for 30 minutes at 37°C, and the medium was then replaced. After cGAMP delivery for the indicated time periods, the cell extracts were analyzed for IRF3 dimerization and for IRF3 phosphorylation and TBK1 phosphorylation by native PAGE and SDS-PAGE, respectively. Graphs show the mean ± s.d., and the data (**A**-**H**) shown are representative of three independent experiments. *P <0.05; **P <0.01 (two-tailed t-test).

We further investigated the role of CYLD in bone marrow-derived macrophages (BMDMs). BMDMs from WT or *Cyld* ^-/-^ mice were stimulated separately by poly(dA:dT) or ISD. Compared with that in WT BMDMs, the induction of IFN-β and IFN-α4 mRNAs was markedly attenuated in *Cyld* ^-/-^ BMDMs (Fig 3A and 3C). We also observed that the induction of IFN-β and IFN-α4 mRNAs was increased in *Cyld* ^-/-^ BMDMs compared with that in WT BMDMs stimulated by poly(I:C) (S3A Fig), which is consistent with the data shown in S1A Fig. Moreover, ELISA demonstrated that the production of IFN-β was impaired upon poly(dA:dT) or ISD stimulation (Fig 3B and 3D) in *Cyld* ^-/-^ BMDMs. Furthermore, the gene expression of IFN-β and IFN-α4 was impaired in *Cyld* ^-/-^ BMDMs after challenge with HSV-1 (Fig 3E). Since IFN-β protects host cells against virus infection, we examined the role of CYLD in restricting HSV-1 infection. Fresh MEFs were pretreated with culture supernatants collected from ISD-stimulated *Cyld*-deficient BMDMs or WT BMDMs, followed by HSV-1 infection. Fresh MEFs pretreated with culture supernatants from *Cyld*-deficient BMDMs were more sensitive to HSV-1 infection (S3B Fig).

IRF3 dimerization/phosphorylation and TBK1 phosphorylation are hallmarks of STING signaling pathway activation. Compared to those in WT BMDMs, these processes were markedly attenuated upon HSV-1 challenge in *Cyld* ^-/-^ BMDMs (Fig 3F). *Cyld* knockout also drastically inhibited the expression of IRF3-responsive genes (*Ifnb* and *Ifna4*) induced by cGAMP (Fig 3G). Consistent with this finding, IRF3 dimerization/phosphorylation and TBK1 phosphorylation triggered by cGAMP were almost abolished in *Cyld* ^-/-^ BMDMs (Fig 3H). In addition, the phosphorylation and dimerization of STING were impaired in *Cyld* ^-/-^ BMDMs compared to those in WT BMDMs (S3C Fig). Taken together, these results indicate that CYLD is an important checkpoint for the DNA-triggered STING-dependent signaling pathway.

### CYLD associates with STING in a ubiquitination-dependent manner

**Fig 4:**
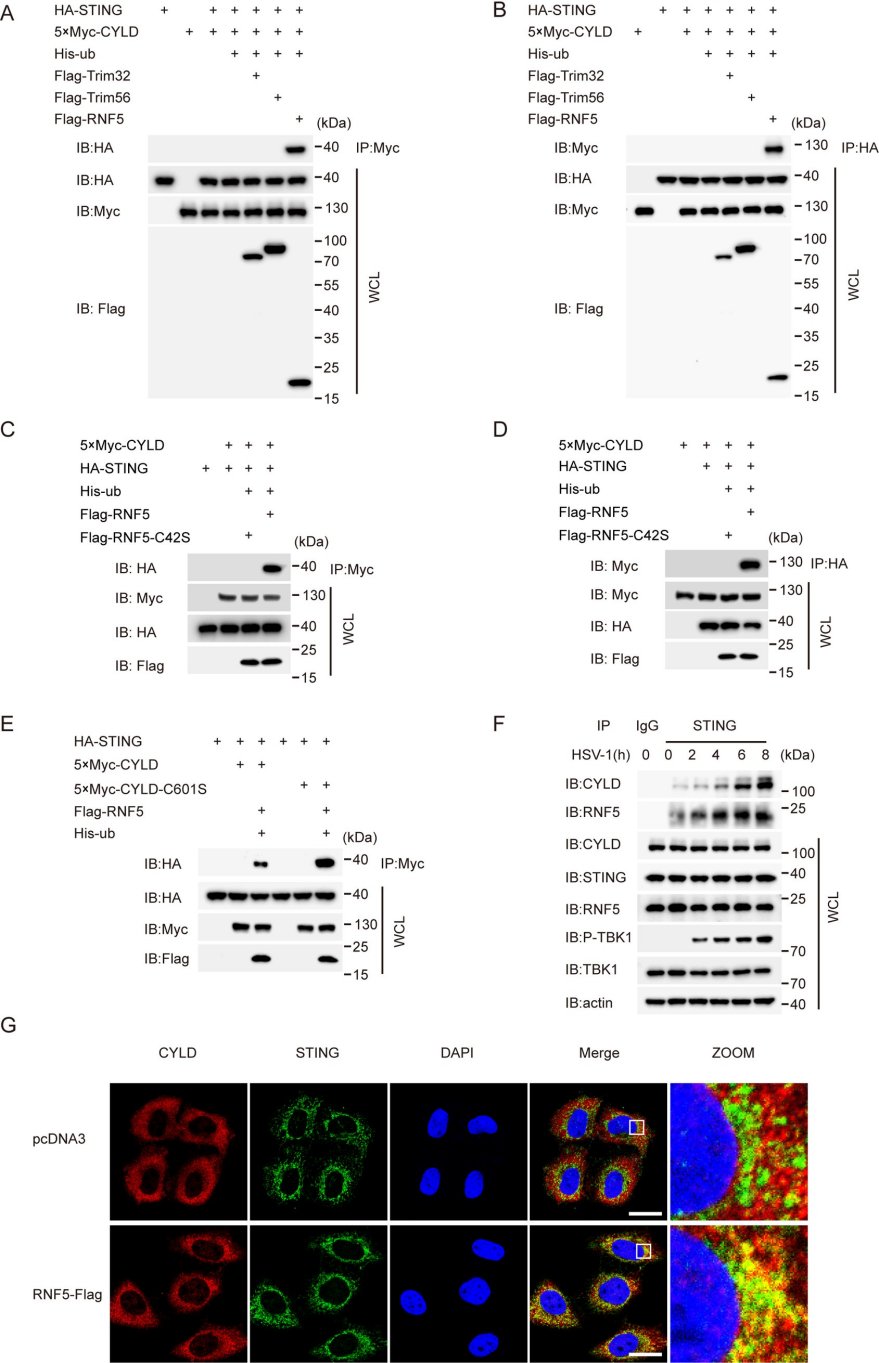
CYLD interacts with STING in a ubiquitination-dependent manner. (**A**) HEK293T cells were transfected with the indicated plasmids. Thirty-six hours after transfection, the cell lysates were immunoprecipitated with an anti-Myc antibody and then immunoblotted with the indicated antibodies. (**B**) HEK293T cells were transfected with the indicated plasmids. Thirty-six hours after transfection, cell lysates were immunoprecipitated with an anti-HA antibody and then immunoblotted with the indicated antibodies. (**C** and **D**) HEK293T cells were transfected with the indicated plasmids. Thirty-six hours after transfection, the cell lysates were immunoprecipitated with an anti-Myc antibody (**C**) or an anti-HA antibody (**D**) and then immunoblotted with the indicated antibodies. (**E**) HEK293T cells were transfected with the indicated plasmids. Thirty-six hours after transfection, the cell lysates were immunoprecipitated with an anti-Myc antibody and then immunoblotted with the indicated antibodies. (**F**) After stimulation with HSV-1 (MOI = 10) for the indicated time periods in the presence of MG132 (10 μM), lysates of MEFs were immunoprecipitated with an anti-STING antibody and then immunoblotted with the indicated antibodies. (**G**) HeLa cells were transfected with empty vector or Flag-tagged RNF5 for 24 h and then stained with the indicated antibodies before imaging by confocal microscopy. STING (Green) (R&D Systems); CYLD (Red) (Abcam); Nucleus (Blue). Scale bars represent 50 μm.

To identify the potential target of CYLD, we expressed the indicated plasmids in HEK293 cells as shown in S4A Fig, which triggered the expression of the IFN-β-luciferase reporter. We observed that the activation of the IFN-β-luciferase reporter by cGAS and STING was markedly reduced when *Cyld* was silenced. In contrast, knockdown of *Cyld* did not affect the activation of the IFN-β-luciferase reporter when TBK1 or IRF3-5D was ectopically expressed. CYLD had a remarkable effect on cGAMP-induced activation of IRF3-responsive genes (Figs 1F, 3G and 3H). Therefore, we wondered whether CYLD could interact directly with STING. Exogenous STING and CYLD were transfected individually or together into HEK293T cells, followed by coimmunoprecipitation (co-IP) assays (Fig 4A and 4B). HA-tagged STING did not interact with Myc-tagged CYLD. It was demonstrated in our previous work that K27-linked polyubiquitin chains on STING can recruit TBK1 and facilitate its translocation to the perinuclear microsomes [21]. We speculated that the polyubiquitin chains of STING may recruit CYLD to STING. To test this possibility, we coexpressed STING and CYLD together with different E3 ligases, namely, RNF5, TRIM32, or TRIM56, in HEK293T cells. RNF5 is known to catalyze K48-linked polyubiquitination of STING [17], while TRIM32 and TRIM56 catalyze the formation of K63-linked polyubiquitin chains on STING [19,20]. Interestingly, the association between CYLD and STING was detected only when the E3 ligase RNF5 was coexpressed. In contrast, the E3 ligase Trim32 or Trim 56 failed to promote the association between STING and CYLD (Fig 4A and 4B). Moreover, a catalytically dead mutant of RNF5 (C42S) did not promote the interaction between STING and CYLD (Fig 4C and 4D). Furthermore, as it was reported that RNF5 ubiquitinated STING at K150 [17], we examined whether lysine 150 of STING could influence the association of STING with CYLD. We coexpressed CYLD and RNF5 with STING or STING K150R and found that the STING K150R mutant failed to associate with CYLD even when RNF5 was present (S4D Fig). Additionally, as shown in S4B Fig, we observed that RNF5 could interact with STING but not CYLD. Recently, TRIM29 was reported to catalyze K48-linked polyubiquitination of STING at lysine 370. TRIM29 is also known to be specifically expressed in airway epithelial cells (AECs) and intestinal epithelial cells [18]. Therefore, we examined if TRIM29 has any effect on the association of STING with CYLD. As shown in S4C Fig, unlike RNF5, TRIM29 could not promote CYLD-STING association. To further investigate if STING and CYLD interact directly, we performed the indicated experiments in S4E Fig. We found that ubiquitinated STING, but not unmodified STING, interacted with CYLD *in vitro*, suggesting that CYLD associates with STING in a ubiquitination-dependent manner. To test whether the catalytically dead CYLD mutant influenced binding with STING, we coexpressed a catalytically dead mutant of human CYLD C601S in the presence of RNF5, as indicated in Fig 4E [34]. As expected, we observed that the human CYLD C601S mutant bound more tightly to STING than the WT human CYLD. A similar result was observed for the catalytically dead murine mutant CYLD C597S (S6A Fig). Then, the endogenous association between STING and CYLD was further investigated. Notably, the endogenous interaction between STING and CYLD was substantially enhanced upon HSV-1 infection (Fig 4F). Consistent with a previous report [17], we also found that RNF5 expression was not changed during HSV-1 infection, while the association of RNF5 with STING was gradually increased. Consistent with this finding, confocal microscopy imaging revealed that the colocalization of STING and CYLD was enhanced in the presence of RNF5 (Figs 4G and S4F). We knew that STING could translocate from the ER to the Golgi upon stimulation with HSV-1, which was also confirmed by our results, as shown in S5A and S5B Fig. Interestingly, we observed that CYLD partially accumulated with STING, which was instrumental for deubiquitinating STING. Taken together, these data establish that CYLD associates with STING in a ubiquitination-dependent manner and colocalizes with STING in the Golgi upon HSV-1 infection.

### CYLD specifically removes K48-linked polyubiquitin chains from STING

**Fig 5:**
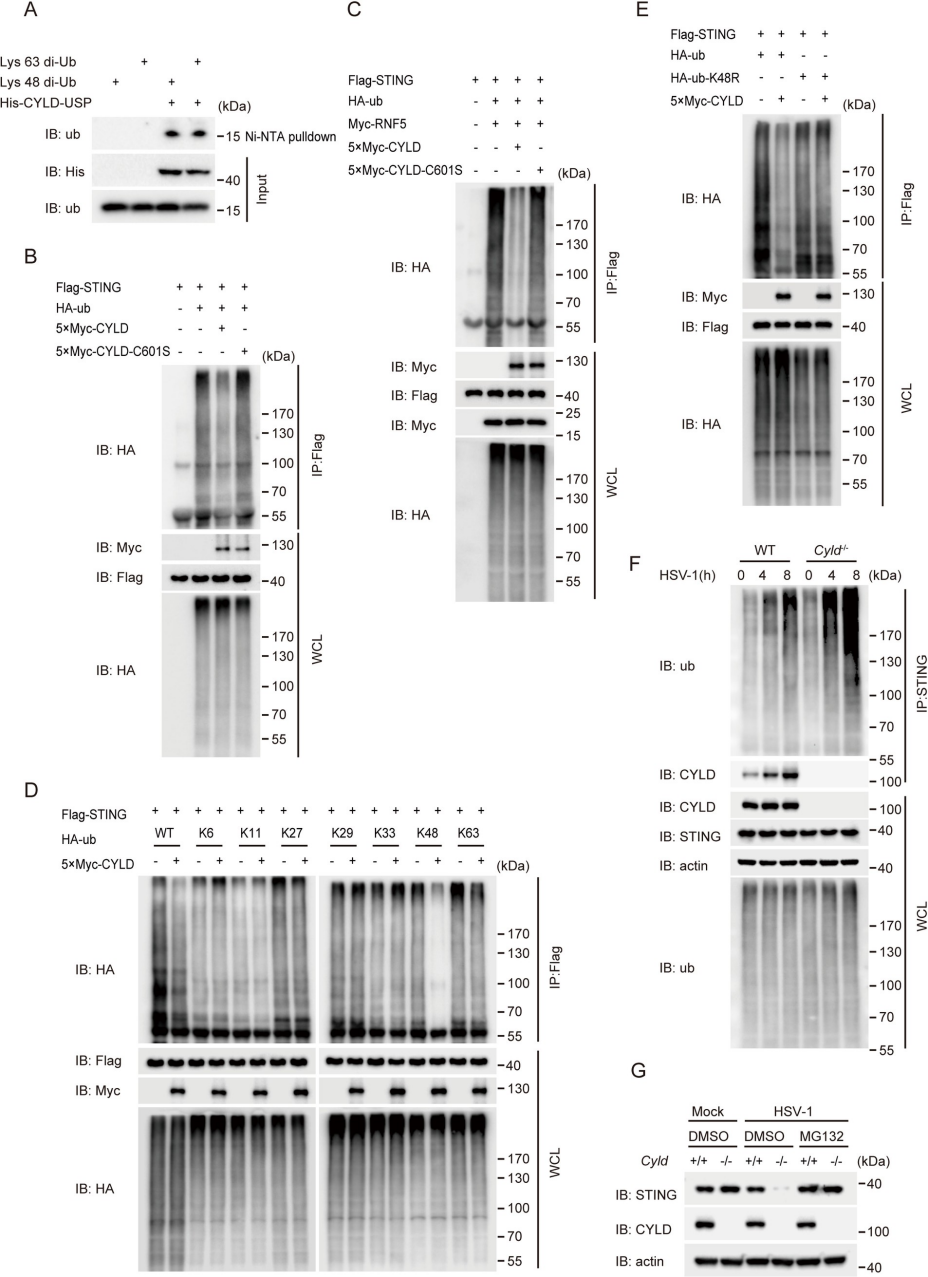
CYLD specifically removes K48-linked polyubiquitin chains from STING. (**A**) K48-linked di-ubiquitin chains (Lys 48-diUb) or K63-linked di-ubiquitin chains (Lys 63-diUb) were subjected to a Ni-NTA pull-down assay with or without His-CYLD-USP (residues 583–956 aa), followed by immunoblotting with the indicated antibodies. (**B**) HEK293T cells were transfected with the indicated plasmids. Thirty-six hours after transfection, the cell lysates were subjected to denaturing immunoprecipitation with an anti-Flag antibody and then analyzed by immunoblotting with the indicated antibodies. (**C**) HEK293T cells were transfected with the indicated plasmids. Thirty-six hours after transfection, the cell lysates were subjected to denaturing immunoprecipitation with an anti-Flag antibody and then analyzed by immunoblotting with the indicated antibodies. (**D**) HEK293T cells were transfected with Flag-STING, 5×Myc-CYLD, HA-tagged-Ub (WT) and various mutants. Thirty-six hours after transfection, the cell lysates were subjected to denaturing immunoprecipitation with an anti-Flag antibody and then analyzed by immunoblotting with the indicated antibodies. (**E**) HEK293T cells were transfected with Flag-STING, 5×Myc-CYLD, HA-tagged-Ub (WT) and a K48R mutant of HA-Ub. Thirty-six hours after transfection, the cell lysates were subjected to denaturing immunoprecipitation with an anti-Flag antibody and then analyzed by immunoblotting with the indicated antibodies. (**F**) WT and *Cyld* ^-/-^ BMDMs were infected with or without HSV-1 (MOI = 10) for the indicated time periods in the presence of MG132 (10 μM). Cell lysates were subjected to denaturing immunoprecipitation with an anti-STING antibody and then analyzed by immunoblotting with the indicated antibodies. (**G**) WT and *Cyld* ^-/-^ BMDMs were infected with or without HSV-1 for the indicated time periods in the presence or absence of MG132 (10 μM) for 6 h, and the cell lysates were analyzed by immunoblotting with the indicated antibodies.

Previous reports demonstrated that CYLD could potentially remove either K63-linked or K48-linked polyubiquitin chains from substrate proteins via its USP domain [27–32]. Notably, the CYLD USP domain appears to preferentially remove K63-linked polyubiquitin chains over other types of polyubiquitin chains [30]. However, it is not known whether CYLD directly binds these chains via its USP domain. Chemically synthesized K63-linked di-ubiquitin chains (K63-diUb) or K48-linked di-ubiquitin chains (K48-diUb) were subjected to pull-down analysis with His-CYLD-USP (residues 583–956 aa). As shown in Fig5A, the His-CYLD-USP domain could directly pull-down both K48-linked and K63-linked di-ubiquitins, supporting previous observations.

We explored whether CYLD could catalyze the deubiquitination of STING. Flag-tagged STING was cotransfected with CYLD or the catalytically dead mutant CYLD C601S. The cell lysates were denatured and subjected to immunoprecipitation with an anti-Flag antibody, followed by immunoblotting, as indicated in Fig 5B. Notably, STING was markedly deubiquitinated in the presence of CYLD, whereas CYLD C601S had no such effect. Consistent with the previous study, RNF5 catalyzed the formation of K48-linked polyubiquitin chains on STING. CYLD efficiently removed the polyubiquitin chains from STING, whereas CYLD C601S failed to do so (Fig 5C). Murine CYLD had the same function as human CYLD (S6B and S6C Fig). We also confirmed that human CYLD and murine CYLD deubiquitinated STING *in vitro*, but the catalytically dead mutants (human CYLD C601S and murine CYLD C597S) could not perform the same function (S6D and S6E Fig). Notably, the CYLD USP domain also deubiquitinated STING *in vitro* (S6D Fig), which is consistent with our observation in S4E Fig. To substantiate this finding, we transfected Flag-STING along with HA-tagged WT ubiquitin or ubiquitin mutants in the presence or absence of CYLD, followed by immunoblotting. As shown in Fig 5D, CYLD selectively removed WT- and K48-linked polyubiquitin chains from STING. Consistent with this result, CYLD failed to remove non-K48-linked polyubiquitin chains (in which lysine 48 of ubiquitin was mutated to arginine) (Fig 5E). These results indicated that CYLD selectively deconjugates the K48-linked ubiquitination of STING. In addition, compared with that in WT BMDMs, endogenous STING polyubiquitination was markedly enhanced in *Cyld* ^-/-^ BMDMs upon HSV-1 stimulation in the presence of the proteasome inhibitor MG132 (Fig 5F). Consistent with this finding, *Cyld* ^-/-^ BMDMs exhibited a marked reduction in the STING protein level upon HSV-1 infection, but this reduction was reversed by MG132 treatment (Fig 5G). Taken together, these data indicate that CYLD prevents STING from undergoing proteasome-mediated degradation by specifically removing K48-linked ubiquitination, thus sustaining STING and promoting the host antiviral response after HSV-1 infection.

### CYLD is required for host defense against HSV-1 infection in mice

**Fig 6:**
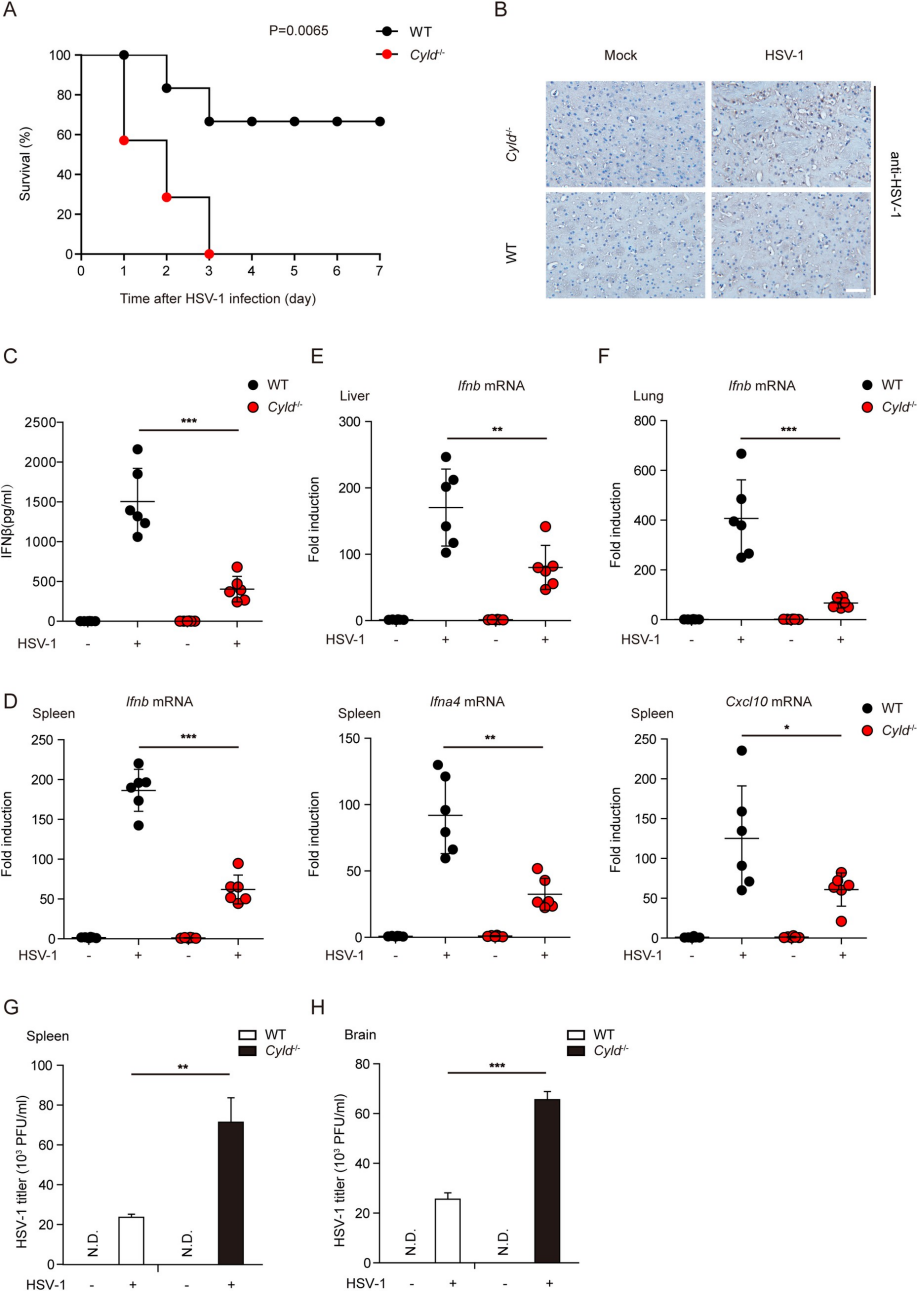
CYLD is required for host defense against HSV-1 infection in mice. (**A**) WT and *Cyld* ^-/-^ mice were injected intravenously with HSV-1 at a dose of 5×10^7^ pfu per mouse, and the survival rates were monitored for 7 days. (**B**) Brains of the HSV-1-infected mice (1×10^7^ pfu per mouse) were harvested for immunohistochemistry (IHC) analysis by an anti-HSV-1 antibody. Tissue sections were visualized by microscopy. Scale bars represent 25 μm. (**C)** WT and *Cyld* ^-/-^ mice were injected intravenously with HSV-1 (1×10^7^ pfu per mouse) for 6 h. Then, the serum was collected, and the concentration of IFN-β was measured by ELISA. (**D**) WT or *Cyld* ^-/-^ mice (n = 6 per group) were injected intravenously with HSV-1 (1×10^7^ pfu per mouse) for 9 h. The relative induction of *Ifnb*, *Ifna4*, and *Cxcl10* mRNAs in spleens from WT or *Cyld* ^-/-^ mice was measured by quantitative PCR. (**E** and **F**) WT or *Cyld* ^-/-^ mice (n = 6 per group) were injected intravenously with HSV-1 (1×10^7^ pfu per mouse) for 9 h. The relative induction of *Ifnb* mRNA in livers (**E**) or lungs (**F**) from WT or *Cyld* ^-/-^ mice was measured by quantitative PCR. (**G** and **H**) WT or *Cyld* ^-/-^ mice (n = 6 per group) were injected intravenously with HSV-1 (1×10^7^ pfu per mouse) for 3 days. The titers of HSV-1 in the spleens (**G**) or brains (**H**) were determined by a standard plaque assay. Graphs show the mean ± s.d., and the data shown are representative of three independent experiments. *P <0.05; **P <0.01; ***P <0.001 (two-tailed t-test).

To address the *in vivo* function of CYLD during infection with a DNA pathogen, WT and *Cyld* ^-/-^ mice were injected intravenously with HSV-1, and their survival rates were monitored. We observed that all *Cyld* ^-/-^ mice died within 3 days, whereas 66.7% of WT mice survived until 7 days after HSV-1 infection (Fig 6A). As expected, HSV-1 virions were more abundant in the brains of *Cyld* ^-/-^ mice than in the brains of WT mice (Fig 6B). Notably, *Cyld* ^-/-^ mice displayed a more severe defect in the production of serum IFN-β after HSV-1 invasion than WT mice (Fig 6C). The expression of *Ifnb*, *Ifna4*, and *Cxcl10* mRNAs was markedly lower in spleen tissues from *Cyld* ^-/-^ mice than in spleen tissues from WT mice (Fig 6D). Consistent with this finding, the expression of IFN-β mRNA was also significantly reduced in liver and lung tissues from *Cyld* ^-/-^ mice (Fig 6E and 6F). We also examined the viral titers in the spleens and brains of *Cyld* ^-/-^ mice, which were much higher than those in WT mice, as indicated by a standard plaque assay, as shown in Fig 6G and 6H.

It is well established that STING is required to initiate effective IFN production when the host is infected by HSV-1 [35,36]. According to several seminal studies, STING KO mice had a higher mortality rate than WT mice upon HSV-1 infection [35–37]. Significant amounts of HSV-1 could be detected in the brains of infected STING KO mice but not in the brains of controls. Mice defective in STING abolished microglia-dependent type I IFN expression upon HSV-1 infection and exhibited increased susceptibility to acute Herpes simplex encephalitis [37]. Additionally, analysis of serum from STING KO mice indicated a profound defect in the production of type I IFNs after HSV-1 infection compared to that of infected control animals. Here, we revealed that CYLD KO mice had similar characteristics after injection of HSV-1. Taken together, our data indicate that CYLD is also required *in vivo* for the effective production of type I IFNs and is essential for sustaining protection against HSV-1 infection.

## Discussion

As the convergence point for monitoring cytosolic aberrant DNAs, STING integrates upstream danger signals to activate TBK1/IRF3, which ultimately induces the production of type I IFNs and proinflammatory cytokines [38]. However, misregulation of STING signaling has been implicated in cancers and autoimmune diseases. Thus, the strength and duration of STING signaling are dynamically modulated in a multilayered and highly ordered manner to maintain immune homeostasis. It is well established that modification of STING by different types of polyubiquitin chains serves to fine-tune STING activity in response to extracellular and intracellular stresses [16]. Similarly, removing polyubiquitin chains from STING acted as an effective feedback mechanism to counterbalance the relevant immune responses. A recent report demonstrated that USP13 could remove K27-linked polyubiquitin chains from STING, thus downregulating STING signaling [39]. It remains unknown how other types of STING polyubiquitination are terminated and how STING activity is sustained during viral infection.

In this study, we characterized the ability of CYLD to specifically deconjugate K48-linked polyubiquitin chains from STING, thus sustaining STING activity and promoting host antiviral responses against DNA viruses. Several lines of evidence substantiate these claims. First, silencing CYLD resulted in a marked decrease in the STING-mediated induction of IRF3-responsive genes, which could be rescued by ectopic expression of WT CYLD but not a catalytically dead CYLD mutant. Second, CYLD enhanced STING signaling via its deubiquitination activity. Third, CYLD knockdown impaired the nuclear translocation of IRF3 and facilitated HSV-1 infection. Fourth, knocking out *Cyld* in BMDMs drastically attenuated STING signaling stimulated by DNA mimics, cGAMP or HSV-1. Fifth, CYLD associated with STING via the K48-linked polyubiquitin chains on STING, and this association appeared to be enhanced upon HSV-1 infection. Sixth, *Cyld* ^-/-^ mice displayed a significant decrease in the production of interferons and cytokines became vulnerable to HSV-1 infection *in vivo*.

CYLD was originally reported as a tumor suppressor and later characterized as a deubiquitinase [23,26]. CYLD can suppress the activation of NF-κB or JNK signaling by removing K63-linked polyubiquitin chains from the signaling complex [27–29,40]. A recent study reported that SDC4 enhances the interaction between CYLD and RIG-I, thus promoting the removal of K63-linked polyubiquitin chains on RIG-I [32]. Importantly, CYLD can also deconjugate K48-linked polyubiquitin chains from some target proteins. For example, CYLD specifically removes K48-linked polyubiquitin chains from TRAF2, thereby preventing the proteasome-mediated degradation of TRAF2 [29]. CYLD can also deconjugate K48-linked polyubiquitin chains on LCK, blocking T cell receptor (TCR) downstream signaling [27]. Our study revealed that CYLD preferentially deconjugates K48-linked polyubiquitin chains on STING, leaving other types of polyubiquitin chains intact. Interestingly, we observed that STING and CYLD colocalized in the Golgi but not in the ER upon HSV-1 stimulation. Therefore, STING translocated from the ER to the Golgi upon HSV-1 stimulation, and CYLD partially accumulated with STING to promote STING deubiquitination. Consequently, CYLD actively prevented the proteasome-mediated degradation of STING and sustained antiviral responses in innate immunity.

Notably, the STING signaling pathway plays a crucial role in dampening cancer development. It is intriguing to consider whether CYLD acts as a tumor suppressor by potentiating STING signaling in cancer. It is also unknown whether human CYLD mutations correlate with the activity of the STING signaling pathway. Insights from these investigations will be instrumental in developing CYLD as a potential target for cancer therapy.

## Materials and methods

### Ethics statement

Animal experiments were carried out in strict accordance with the regulations in the Guide for the Care and Use of Laboratory Animals issued by the Ministry of Science and Technology of the People’s Republic of China. The protocol was approved by the Institutional Animal Care and Use Committee of the Shanghai Institute of Biochemistry and Cell Biology, Chinese Academy of Sciences (Permit Number: IBCB0027 Rev2).

### Mice

*Cyld* knockout mice (in C57BL6/DBA mixed genetic background) were generated and genotyped as described previously [27]. *Cyld* ^+/-^ mice were intercrossed to generate *Cyld* ^+/+^ and *Cyld* ^-/-^ littermates. The mice were maintained under specific pathogen-free (SPF) conditions at the Shanghai Institute of Biochemistry and Cell Biology.

### Cell culture and transfection

HEK293T (ATCC Cat# CRL-11268), HEK293 (ATCC Cat# CRL-1573), HeLa cells (ATCC Cat# CCL-2), MEFs (ATCC Cat#SCRC-1008) were cultured in Dulbecco's modified Eagle's medium (DMEM) plus 10% fetal bovine serum (FBS) (Gibco), supplemented with 1% penicillin-streptomycin (Invitrogen). Vero cells (ATCC Cat# CCL-81) were cultured in MEM (SAFC Biosciences) supplemented with 10% FBS and 1% penicillin-streptomycin. BMDMs (bone marrow-derived macrophages) were prepared as described previously [41]. These cells were maintained in a humidified 5% CO2 incubator at 37˚C. Lipofectamine 2000 (Invitrogen) was used for transfection of plasmid or siRNA according to the manufacturer’s instructions. X-GENE HP from Roche (06 366 236 001) was used for transfection of DNA in MEFs. After incubating the transfection reagent and DNA in a 2:1 (V/M) ratio in the serum-free medium for 15 min, the transfection complex was added to the MEFs for 24 h according to the manufacturer’s instructions.

### Antibodies and reagents

The polyclonal antibody against STING was generated by immunizing rabbit with recombinant human STING (221–379 aa) and also purchased from R&D Systems (MAB7169). The polyclonal antibodies against CYLD were from Abcam (ab137524) and Santa Cruz Biotechnology (sc-74435). The polyclonal antibody against RNF5 was from ABclonal (A8351). The antibody against ER (anti-Calreticulin) was from Abcam (ab2908). The Golgi marker (C10592) was purchased from ThermoFisher. The anti-HSV-1 antibody was from Abcam (ab9533). The antibodies against hemagglutinin (HA), Myc, His, and ubiquitin antibodies were purchased from Santa Cruz Biotechnology. TBK1 antibody was from Abcam (ab40676). IRF3 (D83B9), Phospho-IRF3 (4D4G), Phospho-STING (85735), and Phospho-TBK1 (D52C2) antibodies were from Cell Signaling Technology. Flag and β-actin antibodies were obtained from Sigma-Aldrich. The K48-linked and K63-linked di-ubiquitin were purchased from Boston Biochem. The secondary antibody AlexaFluor 488 (Goat anti-Mouse) and AlexaFluor 647 (Goat anti-Rabbit) were obtained from Molecular Probes. The secondary antibodies Cyanin3 (Goat anti-Mouse; Goat anti-Rabbit) were obtained from Jackson ImmunoResearch.

Wild-type HSV-1 and HSV-1-GFP (F strain) [42] were kindly provided by Dr. Wentao Qiao (Nankai University) and Dr. Chunfu Zheng (Suzhou University), respectively. Poly(dA:dT) was obtained from Sigma-Aldrich. cGAMP was from Invivogen and was delivered into cultured cells by digitonin permeabilization method as previously described [43]. Interferon stimulatory DNA (ISD) was prepared by annealing equimolar amounts of sense and antisense DNA oligonucleotides at 95°C for 10 minutes before cooling to room temperature. Oligonucleotides used as follows:

ISD (sense), 5′-TAC AGA TCT ACT AGT GAT CTA TGA CTG ATC TGT ACA TGA TCT ACA-3′; ISD (antisense), 5′-TGT AGA TCA TGT ACA GAT CAG TCA TAG ATC ACT AGT AGA TCT GTA-3′.

### Plasmids

CYLD, STING, Trim32, Trim56, Trim29 cDNAs were obtained using standard PCR techniques from thymus cDNA library and subsequently inserted into mammalian expression vectors as indicated. The IRF3 5D was a gift from Professor John Hiscott (McGill University, Montreal, Quebec, Canada) [44]. RNF5 and RNF C42S were kindly provided by Dr. Hongbing Shu (Wuhan University)[17]. The CYLD siRNA-resistant form was generated with silent mutants introduced into the siRNA target sequence. All point mutations (CYLD C601S (Human), CYLD C597S (Murine), and STING K150R) were introduced by using a QuickChange XL site-directed mutagenesis method (Stratagene), then were subcloned into the pcDNA3-N-HA vector. The ubiquitin mutants were described previously [21]. All constructs were confirmed by sequencing.

### RNA interference

The siRNAs duplexes were synthesized from Gene-Pharma. The sequences of siRNAs are shown as follows:

*Cyld* siRNA #1 for mouse, 5′-GCU GCU GAA AGU ACC CAA ATT-3′;

*Cyld* siRNA #2 for mouse, 5′-GCA GCC UGU UUC CAA UCA ATT-3′;

*Cyld* siRNA #3 for human, 5′-GGU UCA UCC AGU CAU AAU ATT-3′;

The nonspecific siRNA (N.C.), 5′-UUC UCC GAA CGU GUC ACG UTT-3′.

### Real-time PCR

Total cellular RNA was isolated using TRIzol (Invitrogen) according to the manufacturer’s instructions. Reverse transcription of purified RNA was performed using oligo (dT) primers. The quantifications of gene transcripts were performed by real-time PCR using the FastStart Universal SYBR GREEN MASTER MIX (Roche). Real-time PCR is quantitative compared to *Gapdh* gene and data was normalized to the expression of the gene encoding *Gapdh*. PCR primers of indicated target genes are shown as below:

*Gapdh*: sense (5′-GAA GGG CTC ATG ACC ACA GT-3′),

antisense (5′-GGA TGC AGG GAT GAT GTT CT-3′);

*Ifnb*: sense (5′- AGA TCA ACC TCA CCT ACA GG-3′),

antisense (5′-TCA GAA ACA CTG TCT GCT GG-3′);

*Ifna4*: sense (5′- ACC CAC AGC CCA GAG AGT GAC C-3′),

antisense (5′-AGG CCC TCT TGT TCC CGA GGT-3′);

*Cxcl10*: sense (5′- CGA TGA CGG GCC AGT GAG AAT G-3′),

antisense (5′- TCA ACA CGT GGG CAG GAT AGG CT -3′);

*Cyld*: sense (5′- TCC TGT GAA AGT ACA GCT GC-3′),

antisense (5′- TCC TCA TCA CAC TGG AAA AG -3′);

### Immunoprecipitation assay and immunoblot analysis

For immunoprecipitation assay, cell extracts were prepared by using lysis buffer (50 mM Tris-HCl pH 7.4, 150 mM NaCl, 0.5% Triton X-100, 1 mM EDTA) supplemented with a protease inhibitor cocktail (Roche). The lysate was incubated with appropriate antibodies for 4 hours to overnight at 4°C before adding protein A/G agarose beads for another 2 hours. The beads were washed three times with the lysis buffer and eluted with SDS-loading buffer for 5 minutes.

For denaturing immunoprecipitation, cells were lysed in 1% SDS buffer (50 mM Tris-HCl pH 7.5, 150 mM NaCl, 1% SDS, 10 mM DTT) and denatured by heating for 30 minutes. The lysates were centrifuged and diluted with Lysis buffer (50mM Tris-HCl pH 7.5, 150 mM NaCl, 1 mM EDTA, 1% Triton X-100) until the concentration of SDS was decreased to 0.1%. The diluted lysates were immunoprecipitated with the indicated antibodies for 4 hours to overnight at 4°C before adding protein A/G agarose for 2 hours. After extensive wash, the immunoprecipitates were subjected to immunoblot analysis.

For immunoblot analysis, the immunoprecipitates samples were subjected to SDS-PAGE. The separated proteins were then electrically transfected to PVDF membrane (Millipore). Immunoblotting was probed with indicated primary and secondary antibodies. The protein bands were visualized by using a SuperSignal West Pico chemiluminescence ECL kit (Pierce).

For *in vitro* deubiquitination assays, denature-IP was performed to obtain ubiquitin-modified STING from HEK293T cells cotransfected with Flag-tagged STING and HA-Ubiquitin. The immunoprecipitates were eluted by the Flag peptide (0.3 mg ml^-1^, 60 μl). CYLD, CYLD-C601S, CYLD-USP, mCYLD, and mCYLD-C597S protein were obtained by an *in vitro* transcription and translation kit (Promega). The ubiquitin-modified Flag-STING was incubated with the obtained protein at 37°C for 2 h followed overnight incubation at 16°C in the presence of ATP (1 μM). The mixtures were analyzed by immunoblot with the indicated antibodies.

### Luciferase reporter assay

Cells were seeded in 12-well plates and transfected with reporter gene plasmids (100 ng) combined with siRNAs and other constructs as indicated. The total amount of DNA was kept constant by supplementing with empty vectors. pTK-Renilla reporter plasmid (2 ng) was added to normalize transfection efficiency. Luciferase activity was analyzed with the Dual Luciferase Reporter Assay System (Promega) [45].

### Immunofluorescence and confocal microscopy

Cells seeded onto glass coverslips were fixed for 15 minutes with 4% paraformaldehyde in PBS and permeabilized in 0.25% Triton X-100 in PBS for another 15 minutes, followed by using 5% BSA in PBS for 1 h. Then, cells were stained with indicated primary antibodies followed by incubation with fluorescent-conjugated secondary antibodies. AlexaFluor 488 (Goat anti-Mouse) and Cyanin3 (Goat anti-Rabbit) were used for immunofluorescence in Fig 4G. AlexaFluor 647 (Goat anti-Rabbit) and Cyanin3 (Goat anti-Mouse) were used for immunofluorescence in S5 Fig. The nuclei were counterstained with DAPI (Sigma-Aldrich). Slides were mounted with fluorescent mounting medium (Dako). Images were captured using Leica laser scanning confocal under a 64×oil objective.

### Measurement of cytokines

Concentrations of cytokines in culture supernatants or mouse serum were measured by VeriKine Kit (PBL Assay Science) according to the manufacturer’ s instructions.

### Native PAGE assay

Native gel electrophoresis for IRF3 dimerization was carried out as described previously [46].

### STING dimerization assay

STING dimerization assay was performed as described previously [47]. Briefly, cells were lysed in the lysis buffer (50 mM Tris-HCl pH 7.4, 150 mM NaCl, 0.5% Triton X-100, 1 mM EDTA) with 1 mM NaF and protease cocktail inhibitor. Cell lysates were mixed with sample loading buffer without 2-Mercaptoethanol before loading on the SDS-PAGE electrophoresis. After transferring to the PVDF membrane, 5% milk in TBS with 0.1% Tween 20 (TBST) was used to block the membrane at room temperature for 1 h. STING antibodies were diluted in 5% BSA in TBST, and incubated at 4°C overnight. The membrane was washed with TBST to remove the non-specific binding. Secondary antibody, in 5% milk TBST, was added to the membrane for 1 h at room temperature, the membrane was washed again and imaged by films.

### Histological analysis

Tissues were fixed in 4% paraformaldehyde, embedded in paraffin, cut into sections, and placed on adhesion microscope slides. Sections were subjected to immunohistochemical (IHC) staining according to standard procedures. The anti-HSV-1 antibody (Abcam) was used for staining.

### Recombinant proteins and Ni-NTA pulldown

Recombinant His-CYLD-USP protein was purified from Rosetta (DE3) Escherichia coli by using Ni-nitrilotriacetic acid resin (NTA).

For Ni-NTA pulldown analysis, the purified recombinant His-CYLD-USP protein was incubated with 20 μl Ni-NTA agarose beads (Qiagen) for 2 h at 4°C followed by extensive washing. The preloaded Ni-NTA agarose beads were incubated with di-Ub (K48-linked) or di-Ub (K63-linked) (Boston Biochem), respectively, for 1 h at 4°C. Precipitates were extensively washed and subjected to SDS-PAGE followed by immunoblot analysis.

### Plaque assay

The supernatants of MEFs cultures and the homogenates of brains and spleens from infected mice (or the serial dilutions) were used to infect monolayers of Vero cells. 2 h later, the supernatants or homogenates were removed and the infected Vero cells were washed with pre-warmed PBS twice followed by incubation with a mixed media, which consists of 2×MEM (gibco), 4% FBS, and 2% Low Melting Point Agarose (SIGMA) for another 48 h. Then the cells were fixed with 4% paraformaldehyde for 15 min and stained with 1% crystal violet for 30 min before counting the plaques.

### Statistics

Student’s t-test was used for statistical analysis of two independent treatments. Mouse survival curves and statistics were analyzed with the Mantel-Cox test. P values of less than 0.05 were considered to be statistically significant.

## Supporting information

S1 Fig (Related to Fig 1). CYLD deficiency impairs cGAMP-mediated type I interferon production. (**A**) MEFs transfected with negative control (N.C.) or CYLD siRNAs were stimulated with poly (I:C) (2 μg per well) for 6 h. Then, the induction of *Ifnb*, *Ifna4*, and *Cxcl10* mRNAs was measured by quantitative PCR. (**B**) MEFs were transfected with the indicated siRNA, and the *Cyld* mRNA was measured by quantitative PCR. Graphs show the mean ± s.d., and the data shown are representative of three independent experiments. *P <0.05; **P <0.01 (two-tailed t-test).
(TIF)

S2 Fig (Related to Fig 2). CYLD is essential for HSV-1 restriction. (**A** and **B**) MEFs were transfected with 1 μg of empty vector (EV) or plasmids for the expression of wild-type HA-CYLD or HA-CYLD C597S for 24 h, followed by stimulation with poly(dA:dT) (3 μg per well) or ISD (5 μg per well) for 6 h. Then, the cell lysates were analyzed by immunoblotting with the indicated antibodies. (**C** and **D**) MEFs were transfected with 2 μl of negative control (N.C.) or CYLD siRNA#1 for 24 h and then transfected with the indicated siRNA-resistant constructs for another 24 h, followed by stimulation with poly(dA:dT) (3 μg per well) or ISD (5 μg per well) for 6 h. Then, the cell lysates were analyzed by immunoblotting with the indicated antibodies. (**E**) The amino acid sequence alignment of mouse CYLD and human CYLD. (**F**) MEFs (12-well plate) transfected with negative control (N.C.) or CYLD siRNA#1 were stimulated with poly(dA:dT) (3 μg per well) or ISD (5 μg per well) for 4 h. Then, cell lysates were analyzed by immunoblotting with the indicated antibodies. (**G**) MEFs transfected with the nonspecific control (N.C.) or CYLD siRNA#1 were infected with HSV-1 (MOI = 1) for 6 h. The titers of HSV-1 were determined by a standard plaque assay. Graphs show the mean ± s.d., and the data shown are representative of three independent experiments. **P <0.01 (two-tailed t-test).
(TIF)

S3 Fig (Related to Fig 3). CYLD deficiency enhances RNA-triggered type I IFN expression. (**A**) WT and *Cyld*-deficient BMDMs were stimulated with mock or poly(I:C) (2 μg per well) for the indicated time periods. The induction of *Ifnb* and *Ifna4* mRNAs was measured by quantitative PCR. (**B**) WT and *Cyld* ^-/-^ BMDMs were transfected with mock or ISD (10 μg per well). Equal volumes of culture supernatants from these treatments were applied to fresh MEFs, followed by HSV-1 (MOI = 10) infection. The proliferation of cells was examined by crystal violet staining. Scale bars represent 200 μm. (**C**) WT and *Cyld*-deficient BMDMs were mock infected or infected with HSV-1 (MOI = 5) for the indicated time periods, and the cell extracts were analyzed for STING dimerization and STING phosphorylation. Graphs show the mean ± s.d., and the data shown are representative of three independent experiments. *P <0.05; **P <0.01 (two-tailed t-test).
(TIF)

S4 Fig (Related to Fig 4). Ubiquitinated STING associates with CYLD. (**A**) The negative control (N.C.) or CYLD siRNA#3 was transfected into HEK293 cells together with IFN-β-luciferase and pTK-Renilla reporter plasmids by using Lipofectamine 2000. Forty-eight hours after transfection, the cells were transfected again with cGAS, STING, TBK1 or IRF3-5D by using Lipofectamine 2000 for 16 h before luciferase assays were performed. (**B**) HEK293T cells were transfected with the indicated plasmids. Thirty-six hours after transfection, the cell lysates were immunoprecipitated with an anti-Flag antibody and then immunoblotted with the indicated antibodies. (**C**) HEK293T cells were transfected with the indicated plasmids. Thirty-six hours after transfection, the cell lysates were immunoprecipitated with an anti-HA antibody and then immunoblotted with the indicated antibodies. (**D**) HEK293T cells were transfected with the indicated plasmids. Thirty-six hours after transfection, the cell lysates were immunoprecipitated with an anti-HA antibody and then immunoblotted with the indicated antibodies. (**E**) Flag-STING was transfected into HEK293T cells with or without HA-ubiquitin for 36 h, and cell lysates were immunoprecipitated with an anti-Flag antibody. Then, ubiquitinated STING or STING was eluted from the anti-Flag precipitates by a Flag peptide (0.3 mg ml^-1^, 60 μl). The eluted ubiquitinated-Flag-STING or Flag-STING was subjected to a Ni-NTA pull-down assay with or without recombinant His-CYLD-USP at 37°C for 1 h in the presence of ATP (1 μM). The precipitates were analyzed by immunoblotting with the indicated antibody. **(F**) HeLa cells were transfected with empty vector or Flag-tagged RNF5 for 24 h, and the cell lysates were then immunoblotted with the indicated antibodies. Graphs show the mean ± s.d., and the data shown are representative of three independent experiments. n.s., not significant; **P <0.01 (two-tailed t-test).
(TIF)

S5 Fig (Related to Fig 4). CYLD partially accumulates with STING in the Golgi upon HSV-1 stimulation. (**A**) MEF cells were stimulated with or without HSV-1 (MOI = 1) for 4 h and then stained with the indicated antibodies before imaging by confocal microscopy. STING (Red); ER (Green); CYLD (Purple); Nucleus (Blue). (**B**) MEF cells were stimulated with or without HSV-1 (MOI = 1) for 4 h and then stained with the indicated antibodies before imaging by confocal microscopy. STING (Red); Golgi (Green); CYLD (Purple); Nucleus (Blue). Scale bars represent 25 μm.
(TIF)

S6 Fig (Related to Fig 5). CYLD deubiquitinates STING *in vitro*. (**A**) HEK293T cells were transfected with the indicated plasmids. Thirty-six hours after transfection, the cell lysates were immunoprecipitated with an anti-Myc antibody and then immunoblotted with the indicated antibodies. (**B**) HEK293T cells were transfected with the indicated plasmids. Thirty-six hours after transfection, the cell lysates were subjected to denaturing immunoprecipitation with an anti-Flag antibody and then analyzed by immunoblotting with the indicated antibodies. (**C**) HEK293T cells were transfected with the indicated plasmids. Thirty-six hours after transfection, the cell lysates were subjected to denaturing immunoprecipitation with an anti-Flag antibody and then analyzed by immunoblotting with the indicated antibodies. (**D**) *In vitro* deubiquitination analysis of ubiquitin-modified STING eluted from the denatured IP (anti-Flag) from HEK293T cells transfected with Flag-STING and HA-ubiquitin with Flag peptide, followed by incubation with *in vitro* generated CYLD, CYLD-C601S, and CYLD-USP by an *in vitro* transcription and translation kit. The mixtures were analyzed by immunoblot analysis with the indicated antibodies. (**E**) *In vitro* deubiquitination analysis of ubiquitin-modified mSTING eluted from the denatured IP (anti-Flag) from HEK293T cells transfected with Flag-mSTING and HA-ubiquitin with Flag peptide, followed by incubation with mCYLD and mCYLD-C597S, which were generated by an *in vitro* transcription and translation kit. The mixtures were analyzed by immunoblot analysis with the indicated antibodies.
(TIF)

## References

[1] Takeuchi O, Akira S (2010) Pattern recognition receptors and inflammation. Cell 140: 805–820. 10.1016/j.cell.2010.01.022 20303872

[2] Yoneyama M, Fujita T (2009) RNA recognition and signal transduction by RIG-I-like receptors. Immunol Rev 227: 54–65. 10.1111/j.1600-065X.2008.00727.x 19120475

[3] Barbalat R, Ewald SE, Mouchess ML, Barton GM (2011) Nucleic acid recognition by the innate immune system. Annu Rev Immunol 29: 185–214. 10.1146/annurev-immunol-031210-101340 21219183

[4] Paludan SR, Bowie AG (2013) Immune sensing of DNA. Immunity 38: 870–880. 10.1016/j.immuni.2013.05.004 23706668 PMC3683625

[5] Sun L, Wu J, Du F, Chen X, Chen ZJ (2013) Cyclic GMP-AMP synthase is a cytosolic DNA sensor that activates the type I interferon pathway. Science 339: 786–791. 10.1126/science.1232458 23258413 PMC3863629

[6] Wu J, Sun L, Chen X, Du F, Shi H, et al (2013) Cyclic GMP-AMP is an endogenous second messenger in innate immune signaling by cytosolic DNA. Science 339: 826–830. 10.1126/science.1229963 23258412 PMC3855410

[7] Gao P, Ascano M, Wu Y, Barchet W, Gaffney BL, et al (2013) Cyclic [G(2',5')pA(3',5')p] is the metazoan second messenger produced by DNA-activated cyclic GMP-AMP synthase. Cell 153: 1094–1107. 10.1016/j.cell.2013.04.046 23647843 PMC4382009

[8] Ablasser A, Goldeck M, Cavlar T, Deimling T, Witte G, et al (2013) cGAS produces a 2'-5'-linked cyclic dinucleotide second messenger that activates STING. Nature 498: 380–384. 10.1038/nature12306 23722158 PMC4143541

[9] Takaoka A, Wang Z, Choi MK, Yanai H, Negishi H, et al (2007) DAI (DLM-1/ZBP1) is a cytosolic DNA sensor and an activator of innate immune response. Nature 448: 501–505. 10.1038/nature06013 17618271

[10] Zhang Z, Yuan B, Bao M, Lu N, Kim T, et al (2011) The helicase DDX41 senses intracellular DNA mediated by the adaptor STING in dendritic cells. Nat Immunol 12: 959–965. 10.1038/ni.2091 21892174 PMC3671854

[11] Unterholzner L, Keating SE, Baran M, Horan KA, Jensen SB, et al (2010) IFI16 is an innate immune sensor for intracellular DNA. Nat Immunol 11: 997–1004. 10.1038/ni.1932 20890285 PMC3142795

[12] Ishikawa H, Barber GN (2008) STING is an endoplasmic reticulum adaptor that facilitates innate immune signalling. Nature 455: 674–678. 10.1038/nature07317 18724357 PMC2804933

[13] Zhong B, Yang Y, Li S, Wang YY, Li Y, et al (2008) The adaptor protein MITA links virus-sensing receptors to IRF3 transcription factor activation. Immunity 29: 538–550. 10.1016/j.immuni.2008.09.003 18818105

[14] Jin L, Waterman PM, Jonscher KR, Short CM, Reisdorph NA, et al (2008) MPYS, a novel membrane tetraspanner, is associated with major histocompatibility complex class II and mediates transduction of apoptotic signals. Mol Cell Biol 28: 5014–5026. 10.1128/MCB.00640-08 18559423 PMC2519703

[15] Sun W, Li Y, Chen L, Chen H, You F, et al (2009) ERIS, an endoplasmic reticulum IFN stimulator, activates innate immune signaling through dimerization. Proc Natl Acad Sci U S A 106: 8653–8658. 10.1073/pnas.0900850106 19433799 PMC2689030

[16] Liu X, Wang Q, Pan Y, Wang C (2015) Sensing and responding to cytosolic viruses invasions: An orchestra of kaleidoscopic ubiquitinations. Cytokine Growth Factor Rev 26: 379–387. 10.1016/j.cytogfr.2015.03.001 25862437

[17] Zhong B, Zhang L, Lei C, Li Y, Mao AP, et al (2009) The ubiquitin ligase RNF5 regulates antiviral responses by mediating degradation of the adaptor protein MITA. Immunity 30: 397–407. 10.1016/j.immuni.2009.01.008 19285439

[18] Xing J, Zhang A, Zhang H, Wang J, Li XC, et al (2017) TRIM29 promotes DNA virus infections by inhibiting innate immune response. Nat Commun 8: 945 10.1038/s41467-017-00101-w 29038422 PMC5643338

[19] Zhang J, Hu MM, Wang YY, Shu HB (2012) TRIM32 protein modulates type I interferon induction and cellular antiviral response by targeting MITA/STING protein for K63-linked ubiquitination. J Biol Chem 287: 28646–28655. 10.1074/jbc.M112.362608 22745133 PMC3436586

[20] Tsuchida T, Zou J, Saitoh T, Kumar H, Abe T, et al (2010) The ubiquitin ligase TRIM56 regulates innate immune responses to intracellular double-stranded DNA. Immunity 33: 765–776. 10.1016/j.immuni.2010.10.013 21074459

[21] Wang Q, Liu X, Cui Y, Tang Y, Chen W, et al (2014) The E3 ubiquitin ligase AMFR and INSIG1 bridge the activation of TBK1 kinase by modifying the adaptor STING. Immunity 41: 919–933. 10.1016/j.immuni.2014.11.011 25526307

[22] Qin Y, Zhou MT, Hu MM, Hu YH, Zhang J, et al (2014) RNF26 temporally regulates virus-triggered type I interferon induction by two distinct mechanisms. PLoS Pathog 10: e1004358 10.1371/journal.ppat.1004358 25254379 PMC4177927

[23] Massoumi R (2010) Ubiquitin chain cleavage: CYLD at work. Trends Biochem Sci 35: 392–399. 10.1016/j.tibs.2010.02.007 20347313

[24] Bhoj VG, Chen ZJ (2009) Ubiquitylation in innate and adaptive immunity. Nature 458: 430–437. 10.1038/nature07959 19325622

[25] Sun SC (2008) Deubiquitylation and regulation of the immune response. Nat Rev Immunol 8: 501–511. 10.1038/nri2337 18535581 PMC5763493

[26] Bignell GR, Warren W, Seal S, Takahashi M, Rapley E, et al (2000) Identification of the familial cylindromatosis tumour-suppressor gene. Nat Genet 25: 160–165. 10.1038/76006 10835629

[27] Reiley WW, Zhang M, Jin W, Losiewicz M, Donohue KB, et al (2006) Regulation of T cell development by the deubiquitinating enzyme CYLD. Nat Immunol 7: 411–417. 10.1038/ni1315 16501569

[28] Reiley WW, Jin W, Lee AJ, Wright A, Wu X, et al (2007) Deubiquitinating enzyme CYLD negatively regulates the ubiquitin-dependent kinase Tak1 and prevents abnormal T cell responses. J Exp Med 204: 1475–1485. 10.1084/jem.20062694 17548520 PMC2118606

[29] Xue L, Igaki T, Kuranaga E, Kanda H, Miura M, et al (2007) Tumor suppressor CYLD regulates JNK-induced cell death in Drosophila. Dev Cell 13: 446–454. 10.1016/j.devcel.2007.07.012 17765686

[30] Komander D, Lord CJ, Scheel H, Swift S, Hofmann K, et al (2008) The structure of the CYLD USP domain explains its specificity for Lys63-linked polyubiquitin and reveals a B box module. Mol Cell 29: 451–464. 10.1016/j.molcel.2007.12.018 18313383

[31] Yang Y, Liu M, Li D, Ran J, Gao J, et al (2014) CYLD regulates spindle orientation by stabilizing astral microtubules and promoting dishevelled-NuMA-dynein/dynactin complex formation. Proc Natl Acad Sci U S A 111: 2158–2163. 10.1073/pnas.1319341111 24469800 PMC3926035

[32] Lin W, Zhang J, Lin H, Li Z, Sun X, et al (2016) Syndecan-4 negatively regulates antiviral signalling by mediating RIG-I deubiquitination via CYLD. Nat Commun 7: 11848 10.1038/ncomms11848 27279133 PMC4906230

[33] Friedman CS, O'Donnell MA, Legarda-Addison D, Ng A, Cardenas WB, et al (2008) The tumour suppressor CYLD is a negative regulator of RIG-I-mediated antiviral response. EMBO Rep 9: 930–936. 10.1038/embor.2008.136 18636086 PMC2529351

[34] Trompouki E, Hatzivassiliou E, Tsichritzis T, Farmer H, Ashworth A, et al (2003) CYLD is a deubiquitinating enzyme that negatively regulates NF-kappaB activation by TNFR family members. Nature 424: 793–796. 10.1038/nature01803 12917689

[35] Ishikawa H, Ma Z, Barber GN (2009) STING regulates intracellular DNA-mediated, type I interferon-dependent innate immunity. Nature 461: 788–792. 10.1038/nature08476 19776740 PMC4664154

[36] Li XD, Wu J, Gao D, Wang H, Sun L, et al (2013) Pivotal roles of cGAS-cGAMP signaling in antiviral defense and immune adjuvant effects. Science 341: 1390–1394. 10.1126/science.1244040 23989956 PMC3863637

[37] Reinert LS, Lopusna K, Winther H, Sun C, Thomsen MK, et al (2016) Sensing of HSV-1 by the cGAS-STING pathway in microglia orchestrates antiviral defence in the CNS. Nat Commun 7: 13348 10.1038/ncomms13348 27830700 PMC5109551

[38] Wang Q, Liu X, Zhou Q, Wang C (2015) Cytosolic sensing of aberrant DNA: arming STING on the endoplasmic reticulum. Expert Opin Ther Targets 19: 1397–1409. 10.1517/14728222.2015.1067303 26220155

[39] Sun H, Zhang Q, Jing YY, Zhang M, Wang HY, et al (2017) USP13 negatively regulates antiviral responses by deubiquitinating STING. Nat Commun 8: 15534 10.1038/ncomms15534 28534493 PMC5457515

[40] Zhang M, Wu X, Lee AJ, Jin W, Chang M, et al (2008) Regulation of IkappaB kinase-related kinases and antiviral responses by tumor suppressor CYLD. J Biol Chem 283: 18621–18626. 10.1074/jbc.M801451200 18467330 PMC2441564

[41] Lou X, Sun S, Chen W, Zhou Y, Huang Y, et al (2011) Negative feedback regulation of NF-kappaB action by CITED2 in the nucleus. J Immunol 186: 539–548. 10.4049/jimmunol.1001650 21098220

[42] Li Y, Wang S, Zhu H, Zheng C (2011) Cloning of the herpes simplex virus type 1 genome as a novel luciferase-tagged infectious bacterial artificial chromosome. Arch Virol 156: 2267–2272. 10.1007/s00705-011-1094-9 21894520

[43] Girardin SE, Boneca IG, Carneiro LA, Antignac A, Jehanno M, et al (2003) Nod1 detects a unique muropeptide from gram-negative bacterial peptidoglycan. Science 300: 1584–1587. 10.1126/science.1084677 12791997

[44] Grandvaux N, Servant MJ, tenOever B, Sen GC, Balachandran S, et al (2002) Transcriptional profiling of interferon regulatory factor 3 target genes: direct involvement in the regulation of interferon-stimulated genes. J Virol 76: 5532–5539. 10.1128/JVI.76.11.5532-5539.2002 11991981 PMC137057

[45] Cui Y, Yu H, Zheng X, Peng R, Wang Q, et al (2017) SENP7 Potentiates cGAS Activation by Relieving SUMO-Mediated Inhibition of Cytosolic DNA Sensing. PLoS Pathog 13: e1006156 10.1371/journal.ppat.1006156 28095500 PMC5271409

[46] Iwamura T, Yoneyama M, Yamaguchi K, Suhara W, Mori W, et al (2001) Induction of IRF-3/-7 kinase and NF-kappaB in response to double-stranded RNA and virus infection: common and unique pathways. Genes Cells 6: 375–388. 11318879 10.1046/j.1365-2443.2001.00426.x

[47] Dou Z, Ghosh K, Vizioli MG, Zhu J, Sen P, et al (2017) Cytoplasmic chromatin triggers inflammation in senescence and cancer. Nature 550: 402–406. 10.1038/nature24050 28976970 PMC5850938

