# Supplementary figures and images for: The deubiquitinase CYLD is a specific checkpoint of the STING antiviral signaling pathway
Source: PLoS Pathog. 2018 Nov 2;14(11):e1007435. doi: 10.1371/journal.ppat.1007435 (PMC6235404; doi:10.1371/journal.ppat.1007435)

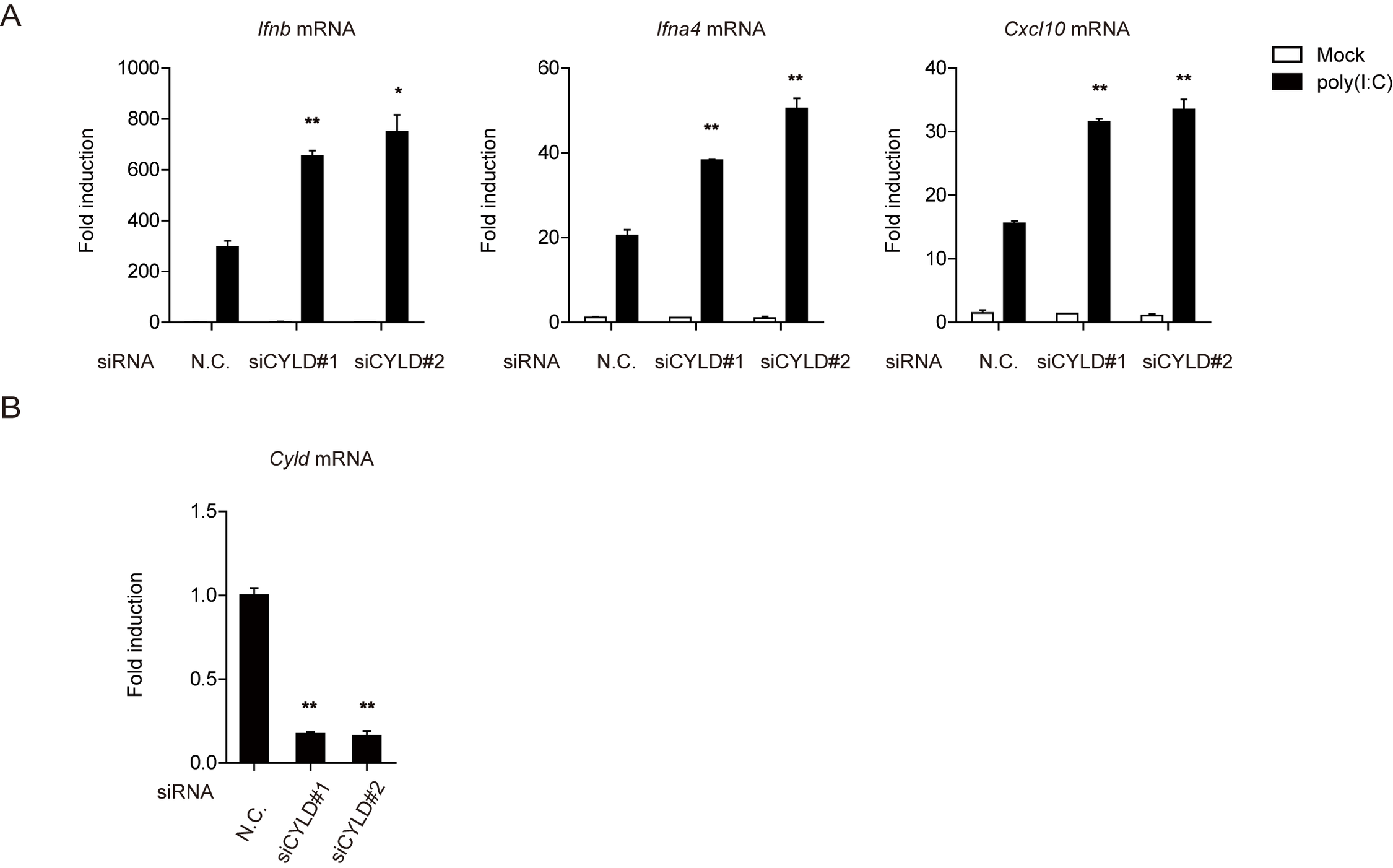

Supplement: S1 Fig — CYLD deficiency impairs cGAMP-mediated type I interferon production. (A) MEFs transfected with negative control (N.C.) or CYLD siRNAs were stimulated with poly (I:C) (2 μg per well) for 6 h. Then, the induction of Ifnb, Ifna4, and Cxcl10 mRNAs was measured by quantitative PCR. (B) MEFs were transfected with the indicated siRNA, and the Cyld mRNA was measured by quantitative PCR. Graphs show the mean ± s.d., and the data shown are representative of three independent experiments. *P <0.05; **P <0.01 (two-tailed t-test). (TIF) [file ppat.1007435.s001.tif]

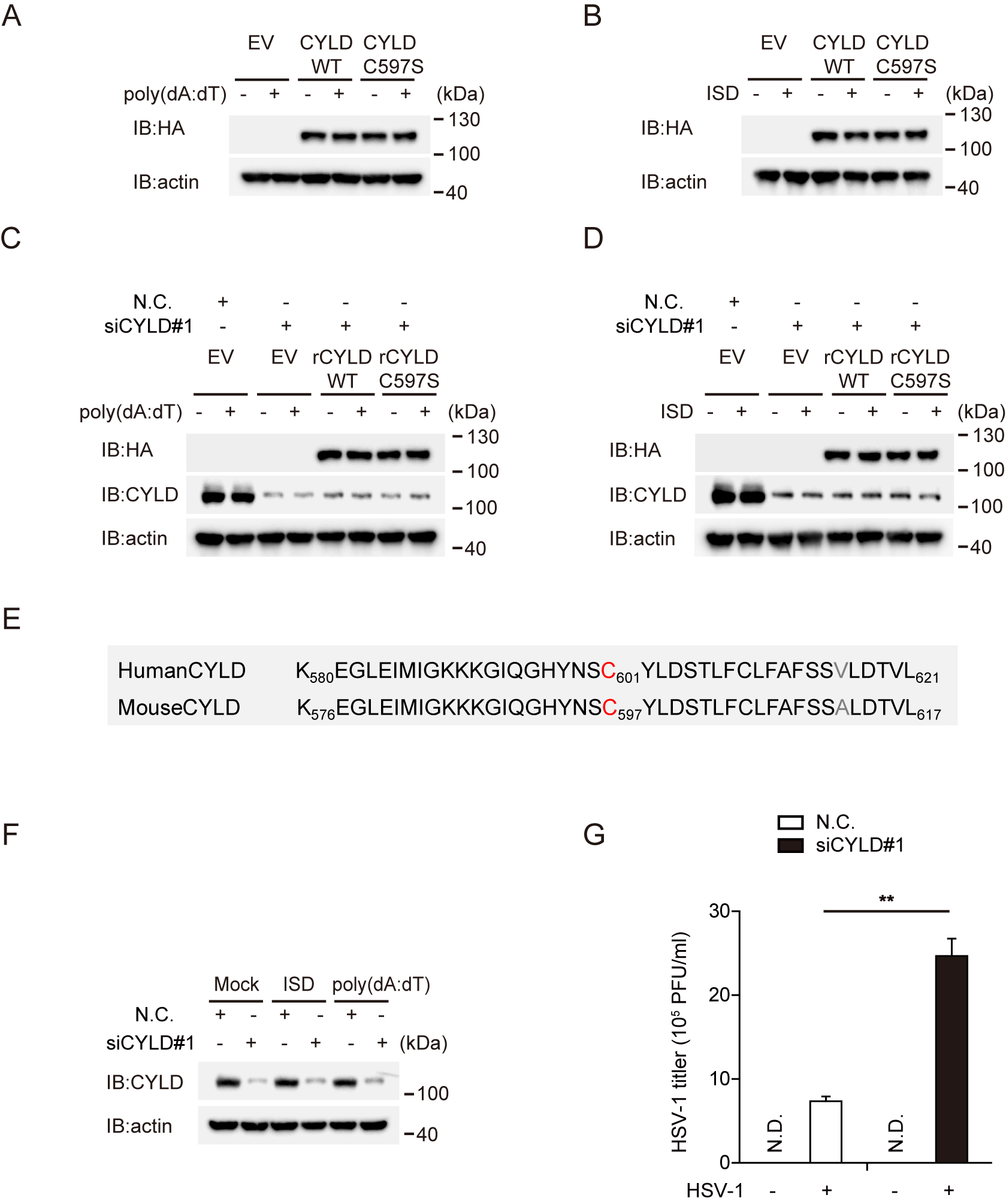

Supplement: S2 Fig — CYLD is essential for HSV-1 restriction. (A and B) MEFs were transfected with 1 μg of empty vector (EV) or plasmids for the expression of wild-type HA-CYLD or HA-CYLD C597S for 24 h, followed by stimulation with poly(dA:dT) (3 μg per well) or ISD (5 μg per well) for 6 h. Then, the cell lysates were analyzed by immunoblotting with the indicated antibodies. (C and D) MEFs were transfected with 2 μl of negative control (N.C.) or CYLD siRNA#1 for 24 h and then transfected with the indicated siRNA-resistant constructs for another 24 h, followed by stimulation with poly(dA:dT) (3 μg per well) or ISD (5 μg per well) for 6 h. Then, the cell lysates were analyzed by immunoblotting with the indicated antibodies. (E) The amino acid sequence alignment of mouse CYLD and human CYLD. (F) MEFs (12-well plate) transfected with negative control (N.C.) or CYLD siRNA#1 were stimulated with poly(dA:dT) (3 μg per well) or ISD (5 μg per well) for 4 h. Then, cell lysates were analyzed by immunoblotting with the indicated antibodies. (G) MEFs transfected with the nonspecific control (N.C.) or CYLD siRNA#1 were infected with HSV-1 (MOI = 1) for 6 h. The titers of HSV-1 were determined by a standard plaque assay. Graphs show the mean ± s.d., and the data shown are representative of three independent experiments. **P <0.01 (two-tailed t-test). (TIF) [file ppat.1007435.s002.tif]

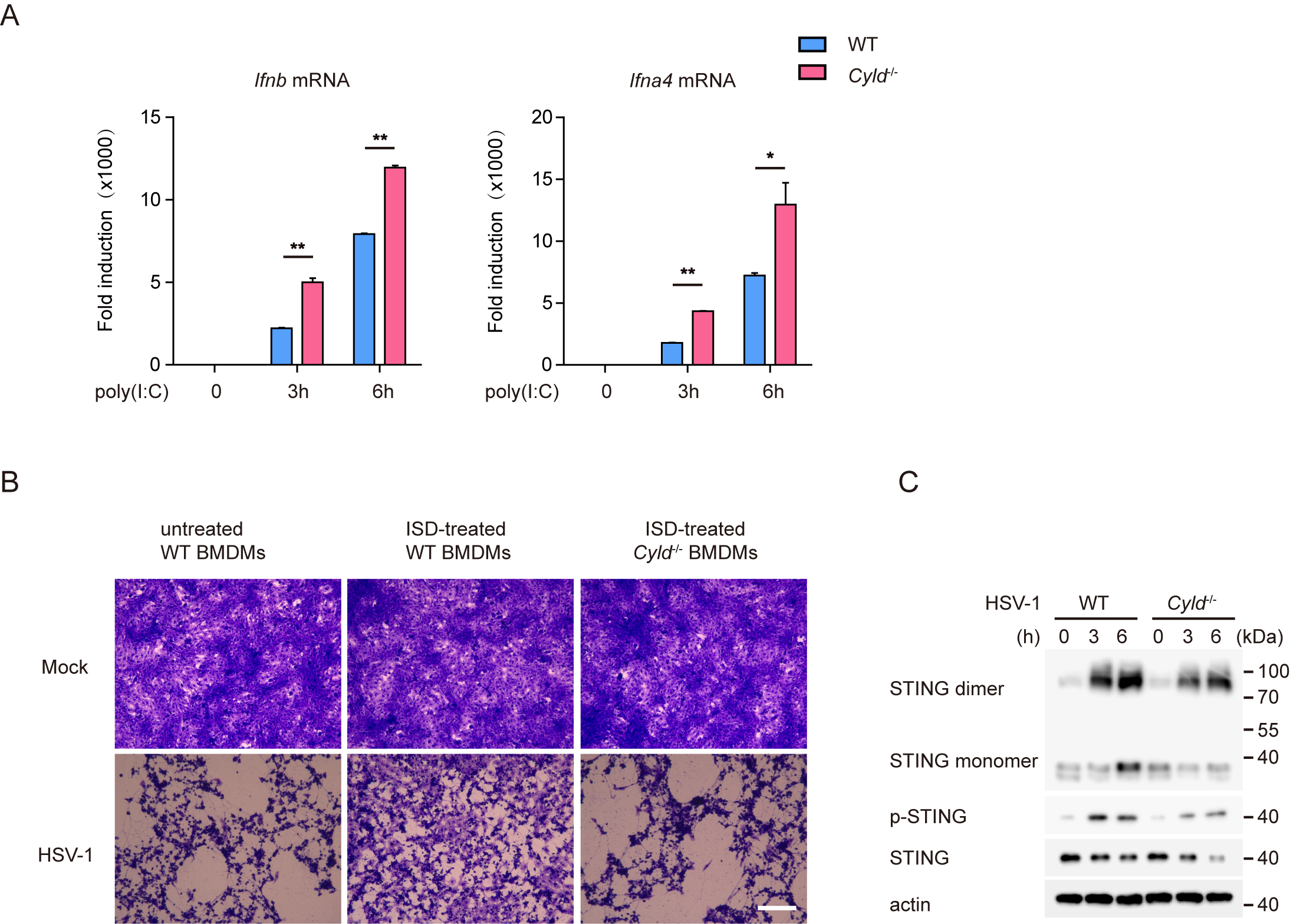

Supplement: S3 Fig — CYLD deficiency enhances RNA-triggered type I IFN expression. (A) WT and Cyld-deficient BMDMs were stimulated with mock or poly(I:C) (2 μg per well) for the indicated time periods. The induction of Ifnb and Ifna4 mRNAs was measured by quantitative PCR. (B) WT and Cyld -/- BMDMs were transfected with mock or ISD (10 μg per well). Equal volumes of culture supernatants from these treatments were applied to fresh MEFs, followed by HSV-1 (MOI = 10) infection. The proliferation of cells was examined by crystal violet staining. Scale bars represent 200 μm. (C) WT and Cyld-deficient BMDMs were mock infected or infected with HSV-1 (MOI = 5) for the indicated time periods, and the cell extracts were analyzed for STING dimerization and STING phosphorylation. Graphs show the mean ± s.d., and the data shown are representative of three independent experiments. *P <0.05; **P <0.01 (two-tailed t-test). (TIF) [file ppat.1007435.s003.tif]

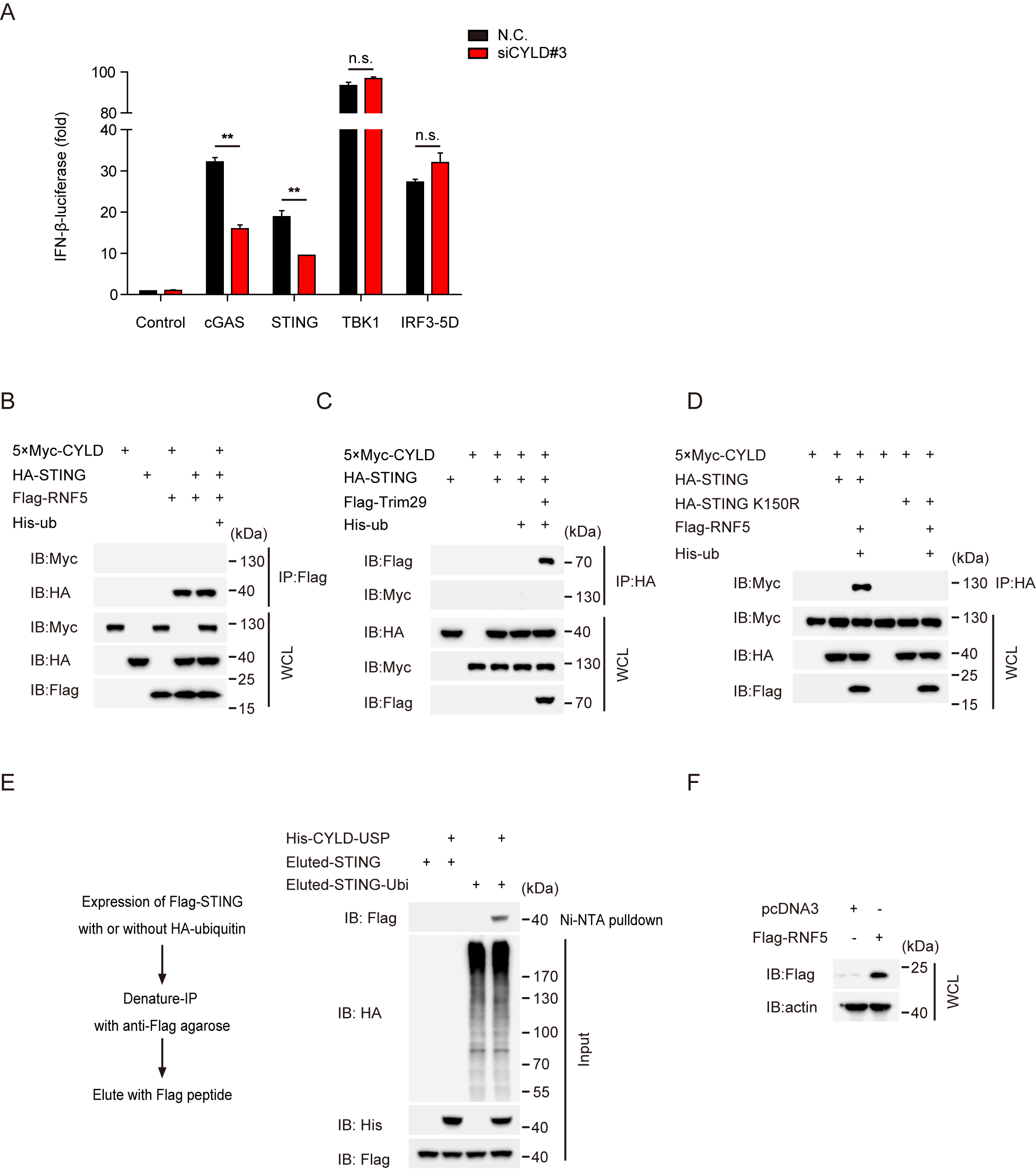

Supplement: S4 Fig — Ubiquitinated STING associates with CYLD. (A) The negative control (N.C.) or CYLD siRNA#3 was transfected into HEK293 cells together with IFN-β-luciferase and pTK-Renilla reporter plasmids by using Lipofectamine 2000. Forty-eight hours after transfection, the cells were transfected again with cGAS, STING, TBK1 or IRF3-5D by using Lipofectamine 2000 for 16 h before luciferase assays were performed. (B) HEK293T cells were transfected with the indicated plasmids. Thirty-six hours after transfection, the cell lysates were immunoprecipitated with an anti-Flag antibody and then immunoblotted with the indicated antibodies. (C) HEK293T cells were transfected with the indicated plasmids. Thirty-six hours after transfection, the cell lysates were immunoprecipitated with an anti-HA antibody and then immunoblotted with the indicated antibodies. (D) HEK293T cells were transfected with the indicated plasmids. Thirty-six hours after transfection, the cell lysates were immunoprecipitated with an anti-HA antibody and then immunoblotted with the indicated antibodies. (E) Flag-STING was transfected into HEK293T cells with or without HA-ubiquitin for 36 h, and cell lysates were immunoprecipitated with an anti-Flag antibody. Then, ubiquitinated STING or STING was eluted from the anti-Flag precipitates by a Flag peptide (0.3 mg ml-1, 60 μl). The eluted ubiquitinated-Flag-STING or Flag-STING was subjected to a Ni-NTA pull-down assay with or without recombinant His-CYLD-USP at 37°C for 1 h in the presence of ATP (1 μM). The precipitates were analyzed by immunoblotting with the indicated antibody. (F) HeLa cells were transfected with empty vector or Flag-tagged RNF5 for 24 h, and the cell lysates were then immunoblotted with the indicated antibodies. Graphs show the mean ± s.d., and the data shown are representative of three independent experiments. n.s., not significant; **P <0.01 (two-tailed t-test). (TIF) [file ppat.1007435.s004.tif]

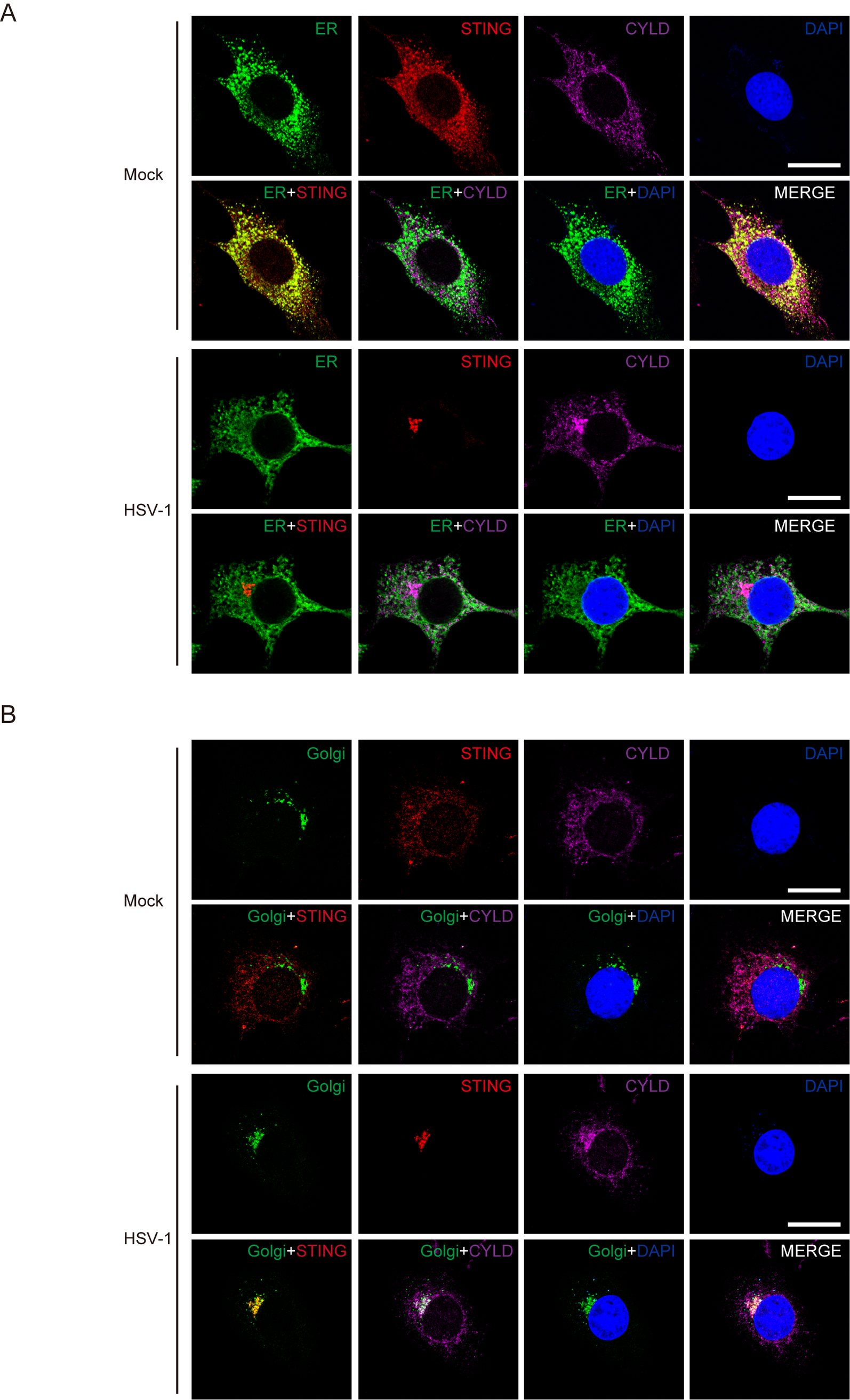

Supplement: S5 Fig — CYLD partially accumulates with STING in the Golgi upon HSV-1 stimulation. (A) MEF cells were stimulated with or without HSV-1 (MOI = 1) for 4 h and then stained with the indicated antibodies before imaging by confocal microscopy. STING (Red); ER (Green); CYLD (Purple); Nucleus (Blue). (B) MEF cells were stimulated with or without HSV-1 (MOI = 1) for 4 h and then stained with the indicated antibodies before imaging by confocal microscopy. STING (Red); Golgi (Green); CYLD (Purple); Nucleus (Blue). Scale bars represent 25 μm. (TIF) [file ppat.1007435.s005.tif]

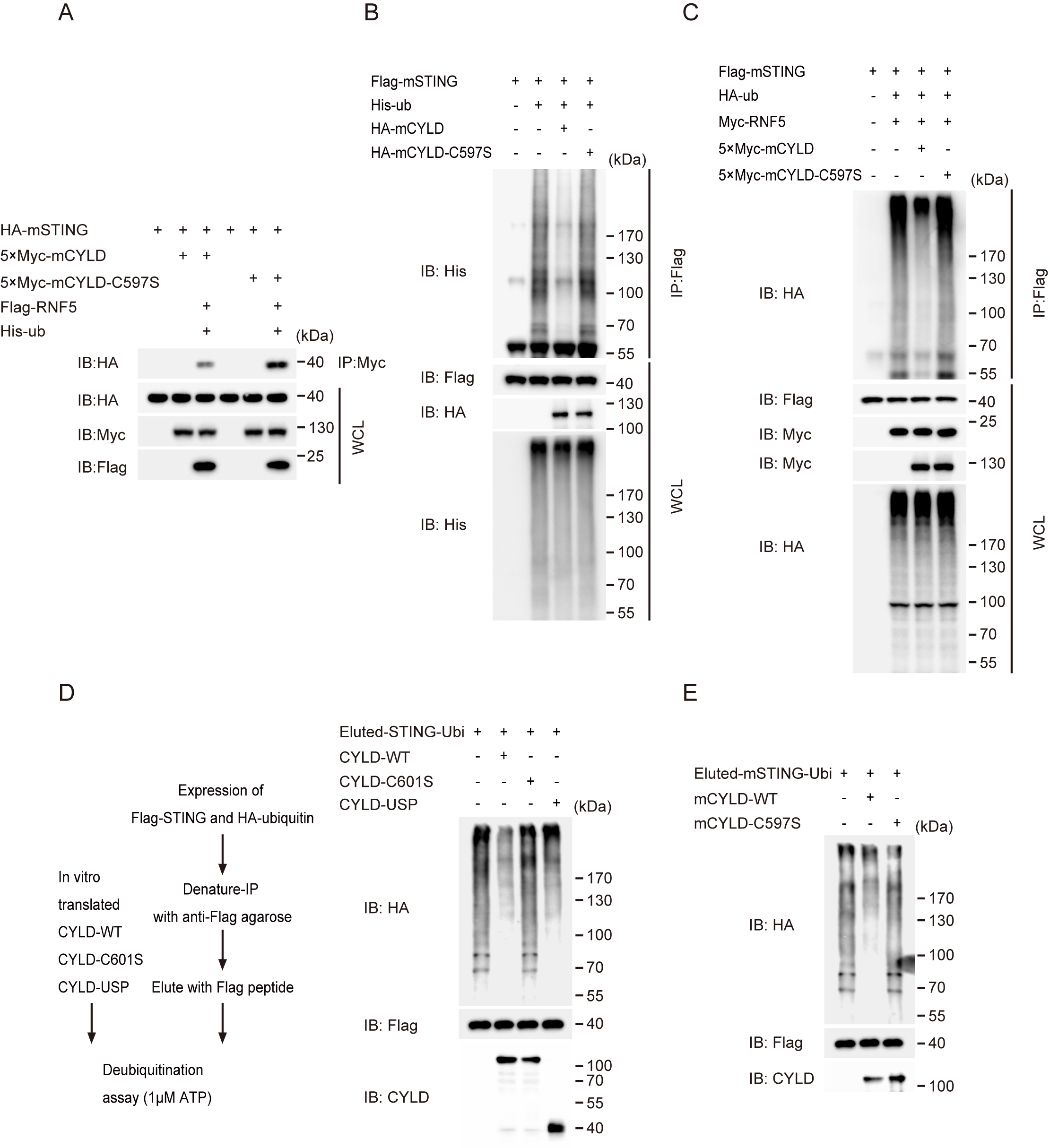

Supplement: S6 Fig — CYLD deubiquitinates STING in vitro. (A) HEK293T cells were transfected with the indicated plasmids. Thirty-six hours after transfection, the cell lysates were immunoprecipitated with an anti-Myc antibody and then immunoblotted with the indicated antibodies. (B) HEK293T cells were transfected with the indicated plasmids. Thirty-six hours after transfection, the cell lysates were subjected to denaturing immunoprecipitation with an anti-Flag antibody and then analyzed by immunoblotting with the indicated antibodies. (C) HEK293T cells were transfected with the indicated plasmids. Thirty-six hours after transfection, the cell lysates were subjected to denaturing immunoprecipitation with an anti-Flag antibody and then analyzed by immunoblotting with the indicated antibodies. (D) In vitro deubiquitination analysis of ubiquitin-modified STING eluted from the denatured IP (anti-Flag) from HEK293T cells transfected with Flag-STING and HA-ubiquitin with Flag peptide, followed by incubation with in vitro generated CYLD, CYLD-C601S, and CYLD-USP by an in vitro transcription and translation kit. The mixtures were analyzed by immunoblot analysis with the indicated antibodies. (E) In vitro deubiquitination analysis of ubiquitin-modified mSTING eluted from the denatured IP (anti-Flag) from HEK293T cells transfected with Flag-mSTING and HA-ubiquitin with Flag peptide, followed by incubation with mCYLD and mCYLD-C597S, which were generated by an in vitro transcription and translation kit. The mixtures were analyzed by immunoblot analysis with the indicated antibodies. (TIF) [file ppat.1007435.s006.tif]
